# Functional Roles for Synaptic-Depression within a Model of the Fly Antennal Lobe

**DOI:** 10.1371/journal.pcbi.1002622

**Published:** 2012-08-23

**Authors:** Aaditya V. Rangan

**Affiliations:** Courant Institute of Mathematical Sciences, New York University, New York, New York, United States of America; Gatsby Computational Neuroscience Unit, University College London, United Kingdom

## Abstract

Several experiments indicate that there exists substantial synaptic-depression at the synapses between olfactory receptor neurons (ORNs) and neurons within the drosophila antenna lobe (AL). This synaptic-depression may be partly caused by vesicle-depletion, and partly caused by presynaptic-inhibition due to the activity of inhibitory local neurons within the AL. While it has been proposed that this synaptic-depression contributes to the nonlinear relationship between ORN and projection neuron (PN) firing-rates, the precise functional role of synaptic-depression at the ORN synapses is not yet fully understood. In this paper we propose two hypotheses linking the information-coding properties of the fly AL with the network mechanisms responsible for ORN
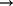
AL synaptic-depression. Our first hypothesis is related to variance coding of ORN firing-rate information — once stimulation to the ORNs is sufficiently high to saturate glomerular responses, further stimulation of the ORNs increases the regularity of PN spiking activity while maintaining PN firing-rates. The second hypothesis proposes a tradeoff between spike-time reliability and coding-capacity governed by the relative contribution of vesicle-depletion and presynaptic-inhibition to ORN
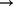
AL synaptic-depression. Synaptic-depression caused primarily by vesicle-depletion will give rise to a very reliable system, whereas an equivalent amount of synaptic-depression caused primarily by presynaptic-inhibition will give rise to a less reliable system that is more sensitive to small shifts in odor stimulation. These two hypotheses are substantiated by several small analyzable toy models of the fly AL, as well as a more physiologically realistic large-scale computational model of the fly AL involving 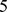 glomerular channels.

## Author Summary

Understanding the intricacies of sensory processing is a major scientific challenge. In this paper we examine the early stages of the olfactory system of the fruit-fly. Many experiments have revealed a great deal regarding the architecture of this system, including the types of neurons within it, as well as the connections those neurons make amongst one another. In this paper we examine the potential dynamics produced by this neuronal network. Specifically, we construct a computational model of this early olfactory system and study the effects of synaptic-depression within this system. We find that the dynamics and coding properties of this system depend strongly on the strength, and sources of, synaptic-depression. This work has ramifications for understanding the coding properties of other insect olfactory systems, and perhaps even other sensory modalities in other animals.

## Introduction

The early stages of the drosophila olfactory system include a primary sensory structure called the antenna lobe (AL). The AL receives input from olfactory sensory neurons (ORNs) at the sensory periphery, and is organized into glomerular clusters, with each cluster corresponding to a specific olfactory receptor class [1]–[5]. Each glomerulus within the AL contains dendrites of local neurons (LNs) whose projections are limited to the AL, as well as projection neurons (PNs) whose axons extend beyond the AL deeper into the fly brain [6]. The PNs are excitatory, whereas there is evidence that both excitatory local neurons (LNEs) and inhibitory local neurons (LNIs) exist [7]–[9]. The LNs associated with each glomerulus have local projections, which connect to that glomerulus, as well as lateral projections which connect to other glomeruli [10].

Various experiments indicate that there exists substantial synaptic-depression at the synapses between olfactory receptor neurons (ORNs) and neurons within the drosophila antenna lobe (AL); by ‘synaptic-depression’, we refer to any mechanism which gives rise to short-term depression of the ORN-induced EPSCs within the AL following an increase in ORN activity. While it has been proposed that this synaptic-depression contributes to the nonlinear relationship between ORN and PN firing-rates, the precise functional role of synaptic-depression at the ORN synapses is not yet fully understood. To investigate the relationship between synaptic-depression and the coding properties of the fly AL, we created and analyzed the dynamics of several models of the fly AL. We have been able to distill two hypotheses linking the information-coding properties of the fly AL with the network mechanisms responsible for ORN
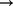
AL synaptic-depression.

Our first hypothesis is related to the variance coding of ORN firing-rate information — once stimulation to the ORNs is sufficiently high to saturate PN responses within any particular glomerular channel, further stimulation of the ORNs can reduce the amount of fluctuation of the ORN
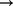
PN input within that channel, thus increasing the regularity of PN spiking activity while maintaining PN firing-rates. Thus, given two different stimuli which saturate the responses of a given glomerulus, it may still be possible to distinguish between these two stimuli solely by using this saturated glomerulus' activity. In order to distinguish these saturated responses, a readout mechanism must be sensitive to higher-order statistics (such as variance) in the saturated glomerulus' activity.

Our second hypothesis proposes a tradeoff between trial-to-trial reliability and sensitivity governed by the mechanisms responsible for ORN
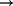
AL synaptic-depression. Within the fly, synaptic-depression may be partly caused by vesicle-depletion, and partly caused by presynaptic-inhibition due to the activity of inhibitory local neurons within the AL [11], [12]. Our second hypothesis is that synaptic-depression caused primarily by vesicle-depletion will give rise to a very reliable system, whereas an equivalent amount of synaptic-depression caused primarily by presynaptic-inhibition will give rise to a less reliable system that is more sensitive to small shifts in odor stimulation. Using this second hypothesis, one can further postulate that a balance of vesicle-depletion and presynaptic-inhibition within the AL is required in order to optimize the discriminability of the network over short observation-times.

## Results

The relationship between the architecture of the fly AL and its odor-coding properties largely remain a mystery. Specifically, the precise functional role of synaptic-depression at the ORN synapses is still unclear. In order to investigate the possible function associated with these network mechanisms, we have designed and built a scaled down computational network model of the fly AL. By analyzing the dynamics of this model we have been able to distill two hypotheses linking the information-coding properties of the fly AL with the network mechanisms responsible for ORN
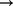
AL synaptic-depression. We will discuss these hypotheses later in the sections below, after first introducing a few pertinent details regarding our computational model.

### Sketch of computational network model

**Figure 1:**
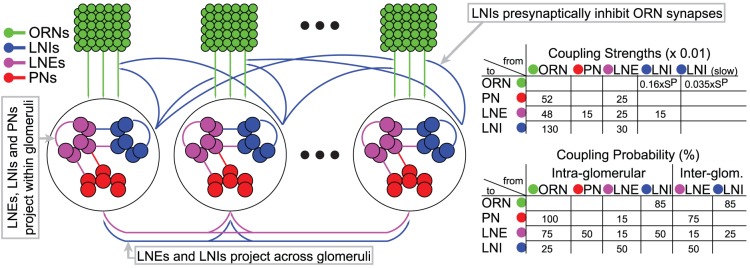
A schematic of the large-scale network model. [Left]: The network consists of 5 glomerular channels, each incorporating 60 olfactory receptor neurons (ORNs in green) which stimulate a ‘glomerulus’ consisting of 6 projection neurons (PNs in red), 6 excitatory local neurons (LNEs in magenta) and 6 inhibitory local neurons (LNIs in blue). The PNs, LNEs and LNIs are connected to one another randomly within each glomerulus, and the LNEs and LNIs also affect the neurons in other glomeruli. The LNIs affect the ORN
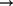
AL synapses via presynaptic-inhibition. [Right]: The non-negligible connection strengths are listed on top, with the slow-inhibitory connection strengths listed separately from the fast-inhibition strengths. The relevant connection probabilities are listed on the bottom. The parameter 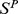 refers to 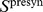, which characterizes the overall strength of presynaptic-inhibition. See Methods for full details.

In brief, our computational network model incorporates 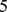 glomerular channels, each with 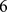 PNs, 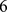 LNEs, 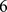 LNIs and 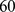 ORNs, in rough accordance with the experimentally observed ratio of ORNs to PNs and LNs [13]. As the real fly AL has 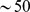 glomerular compartments, each of roughly this size [10], this model is 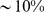 the size of the full AL. Each neuron in this network model is modeled using Hodgkin-Huxley-type equations. The synaptic currents in this network allow neurons to affect other neurons in the same glomerulus, as well as neurons in other glomeruli. The input to this network takes the form of noisy stimulus current to the ORNs, with different ‘odors’ corresponding to different levels of stimulus current to different ORN input channels. Importantly, the model is built to accommodate synaptic-depression of the ORN synapses, allowing for both the mechanisms of presynaptic-inhibition as well as vesicle-depletion. An illustration of the network's connectivity, as well as an abridged list of network parameters, is given in Fig. 1. We have built this network to respect physiological constraints, and we have tuned this model using several experiments as benchmarks. Here we provide a brief summary of these results. A more detailed description of the model as well as the details regarding the benchmarking are contained in the Methods section.

Our goal while benchmarking this model was to ensure that our model produced reasonable statistical features of AL activity during the 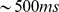 following odor onset. The reason we focused on matching the statistics of this transient period is that evidence indicates that this period is likely critical for many basic olfactory discrimination and classification tasks [14], [15]. One of the simplifications we have made in our model is that the input to the ORNs following odor onset is assumed to be a Poisson process with a time-varying rate that is roughly stereotyped across ORN classes (see Methods). While natural odor stimuli are likely temporally complex [16] and even static stimuli generate odor-specific temporal fluctuations at the level of the fly ORNs after several hundred ms [17], the dynamics of the ORN responses during the first 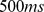 following odor onset seems to be relatively stereotypical, involving either a sharp increase in activity or, more rarely, an inhibitory phase [18], [19]. Thus, the idealized input to the ORNs we employ in our model is intended to capture these simple features of ORN activity which drive the AL during the first 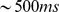 following odor onset.

The experimental phenomena we used to benchmark our model ultimately provided three constraints on the connectivity of our model network. First, the convergence ratio of ORNs to PNs must be high, otherwise the PNs do not receive sufficient convergent input to fire quickly after odor onset. Second, the synaptic-depression at the ORN synapses must be sufficient to ensure that PN firing-rates peak earlier than ORN firing-rates (in response to odor stimulus), and that ORN
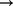
PN input is strong and relatively stable during the first 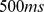 after odor stimulus onset. Finally, the inter-AL connectivity (governed by the LN
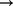
LN, PN
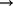
PN, PN
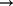
LN, and LN
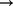
PN connection matrix) must be sufficiently strong to create PNs which are more broadly responsive than their ORN inputs, yet sufficiently sparse to place the network in a dynamic regime which does not develop spontaneous oscillations (which are not observed experimentally during the initial transient following odor onset – [17]).

In addition, to further understand the network mechanisms underlying the two proposed hypotheses, we have designed simpler neuronal network models which distill the relevant phenomena, while allowing for a more comprehensive analysis. The analytical tools we use include the analysis of return-maps for simple network models, as well as the analysis of population-dynamics equations for more complicated network models (see the sections to follow for more details).

### Hypothesis 1: a monotonically decreasing map between ORN activity and PN input variance

As evidenced in [20], [21], the relationship between ORN firing-rate (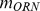) and PN firing-rate (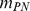) for a given glomerulus is often nonlinear, with the PN firing-rate saturating rather quickly as a function of ORN firing-rate. One consequence of this nonlinearity is that, for low 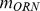, the gain in 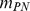 is high — as 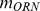 varies from 0–50 Hz, 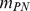 can vary from 0–150 Hz or more. Another consequence of the nonlinear relationship is that, for high 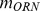, the gain in 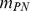 is low — as 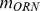 varies from 100–200 Hz, 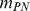 may remain almost constant. Many have noted that the region of high gain allows for ‘odor separation’ — namely, odors which give rise to similar 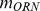 profiles for a given glomerulus may in turn produce very different 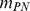 profiles within that glomerulus [20]. However, this ‘odor separation’ only works when the odors in question generate 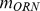 which are sufficiently low as to lie in the region of high 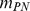 gain. It is tempting to conclude that if two odors generate 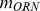 which are sufficiently high (such that the induced 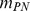 lie in the region of low gain), then the 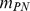 generated by these odors would be similar, and the odors would not be ‘separated’.

The first hypothesis we propose is that, even if two odors generate 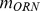 which correspond to similar 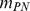, the dynamics of the glomerulus may still serve to separate these odors. However, in this case the odor separation takes place not in terms of PN firing-rates (as, indeed, the 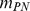 generated by these two odors may be very similar or identical), but rather in terms of higher-order statistics of PN activity. In other words, even though the set of PN firing-rates produced at the plateau of the 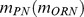 relationship are similar, we hypothesize that there is in fact a systematic difference in the PN dynamics underlying these similar PN firing-rates.

To be more specific, we claim that for values of 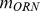 along the plateau of the 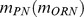 relationship, as 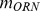 increases (and 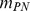 stays roughly the same), the synaptic-depression at the ORN synapses continues to increase. One consequence of this increase in synaptic-depression is that, as 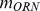 increases along the plateau of 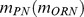, the number of ORN firing-events increases, but the effect of each ORN firing-event on postsynaptic PNs decreases. Thus, the postsynaptic conductance induced within any PN by the ORNs (i.e., the ORN input to the PN) maintains roughly the same mean, but decreases in variance. When discussing a reduction in the variance of ORN input, we refer specifically to a reduction in the variance across short time-windows of the PN excitatory-conductance due to ORN activity.

If the 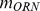 is not very high, then each ORN generates relatively few spikes, each resulting in a large EPSC in the postsynaptic PN. Thus, the ORN input to the PNs will have large fluctuations (i.e., the PNs will be ‘fluctuation-driven’). On the other hand, if 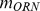 is very high, then each ORN generates very many spikes, each resulting in a small EPSC within the postsynaptic PN. In this case the PN conductance due to the ORNs will be nearly constant (and the PNs will be ‘mean-driven’). We further hypothesize that, as 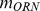 increases along the plateau of 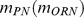, the decrease in variance of ORN input to the PNs will correspond to a decrease in the variance of PN spiking activity. Because (i) the ORN activity is not deterministic, but rather driven by many independent stochastic molecular binding events [18], and (ii) many ORNs are presynaptic to each PN, the accumulation of ORN firing-events observed by any given PN during any trial of odor presentation is well-approximated by a Poisson process with time-varying rate. Thus, a decrease in the ORN input variance across short time-windows will be associated with a decrease in the ORN input variance across multiple trials (for the same time-window). Thus, one would expect the variance in PN spiking activity mentioned above to decrease both across short time-windows and across multiple trials (for the same time-window). This reduction in variance of PN spiking activity is equivalent to an increase in the regularity of PN spiking activity, which is equivalent to a reduction in the variance of the inter-spike-interval distribution associated with a PN within the given glomerulus.

Thus, in summary, our first hypothesis is that the dynamics of a glomerulus can serve to separate ORN inputs in two ways. Not only can similar ORN inputs within the high-gain region of 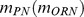 be mapped to significantly different PN firing-rates (see [20]), but ORN inputs within the low-gain region of 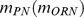 can give rise to PN activity with differing degrees of regularity, even when the PN firing-rates associated with those ORN inputs are not significantly different. This hypothesis may have significance for odor discrimination, as the variance in PN activity may encode features of the odor even in situations where the ORN input is sufficiently high that PN firing-rates have saturated (see Discussion).

#### A simple cartoon of variance coding

As a simple cartoon which illustrates this hypothesis, we have simulated a single conductance-based integrate-and-fire PN, driven by a set of 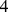 ORNs, each endowed with a simple model of synaptic-depression. This simple model exhibits the following dynamical features: (i) the 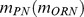 relationship exhibits high gain and saturation, and (ii) for different values of 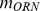 on the plateau of the 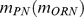 relationship, the variance in PN activity decreases as 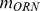 increases, even though 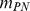 remains roughly constant.

Within this simple model, we describe each ORN as a Poisson process with fixed rate 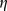 (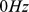 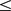 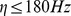). The coupling strength 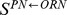 between the ORNs and the PN is modulated by a term 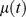 (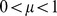), which is intended to model vesicle-depletion at the ORN synapses. As each ORN fires, this 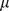 term will give rise to synaptic-depression between the ORNs and the PN. If 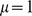, the synapses between the ORNs and the PN are 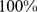 exhausted. If 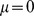, the synapses between the ORNs and the PN are completely refreshed. The model details are given in a section entitled “An idealized model used to illustrate variance coding” in Methods.

**Figure 2:**
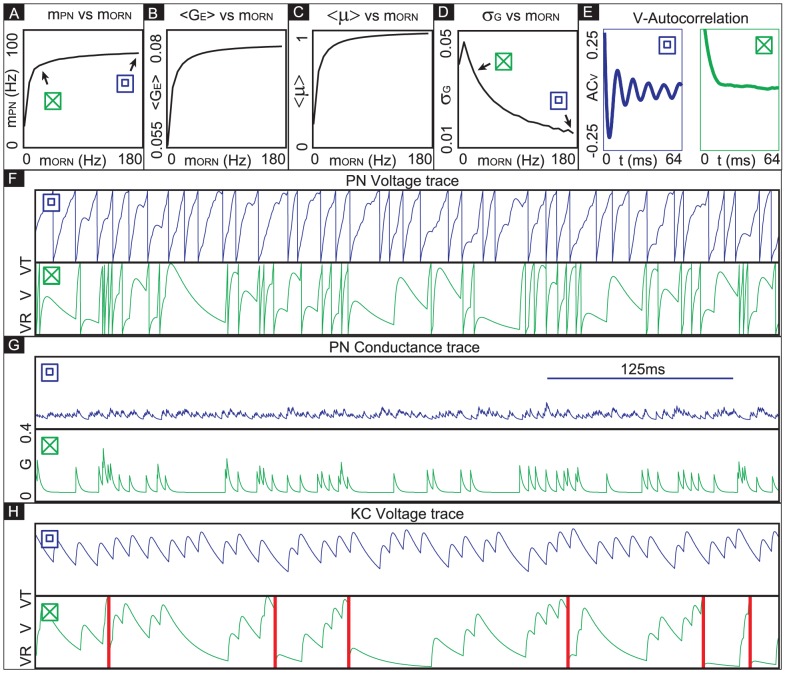
A simple illustration of variance coding. Here we presume the simple model described in the section entitled “An idealized model used to illustrate variance coding”. [A] There is a nonlinear relationship between the ORN firing-rate and the PN firing-rate. [B] There is also a nonlinear relationship between the ORN firing-rate and the time-averaged conductance of the PN. [C] As the ORN firing-rate increases, the time-averaged vesicle-depletion parameter increases and saturates. [D] Since the average vesicle-depletion parameter increases as the ORN firing-rate increases, the variance in the PN conductance is a decreasing function of ORN firing-rate, for sufficiently high ORN firing-rates. Two different points along this curve are indicated, corresponding to two different PN dynamical regimes with similar PN firing-rates. The ‘
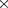
’ and ‘
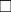
’ symbols indicate, respectively, an irregularly firing-regime and a regularly firing-regime. [E] As a result of the fact that the PN conductance has a low variance when the ORN firing-rates are high, the PN activity is very regular when the ORN firing-rate is high. In contrast, the PN activity is less regular when the ORN firing-rate is not as high. This is reflected in the normalized PN autocorrelation, which shows several significant peaks when the variance in the PN conductance is low (‘
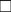
’-regime, left). In contrast, when the variance in the PN conductance is high the autocorrelation does not show significant peaks (‘
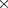
’-regime, right). [F] The regularity in the PN spiking activity is seen in PN voltage trace, as shown for the ‘
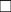
’-regime (top) and ‘
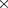
’-regime (bottom). [G] The variance in the PN conductance is seen in PN conductance trace, as shown for the ‘
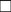
’-regime (top) and ‘
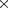
’-regime (bottom). [H] In this panel we show the voltage-trace of a putative Kenyon cell, a conductance-based integrate-and-fire-neuron, driven by either the PN from the 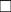-regime (top) or the PN from the 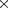-regime (bottom). Thick vertical lines indicate firing-events for this putative KC. When driven by the regular activity of the 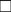-PN, the KC mainains an elevated subthreshold voltage, but does not fire often. On the other hand, when driven by the irregular activity of the 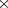-PN, the KC does not maintain an elevated subthreshold voltage but fires after each burst in 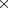-PN-activity. This provides a simple illustration of one possible way in a variance-code could be ‘read-out’ by downstream neurons.

With this simple model, it can be seen that the PN firing-rate 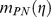 is a nonlinear function of the ORN firing-rate 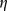, and that 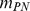 saturates (plateaus) at values of 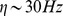 (See Fig. 2A). The time-averaged mean total excitatory conductance 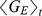 of the PN enjoys a similar nonlinear relationship (Fig. 2B). Notably, for values of 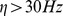, the time-averaged mean vesicle-depletion parameter 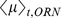 increases as a function of 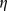, and the standard deviation in the total PN conductance 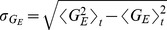 decreases as a function of 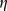 (Fig. 2C and Fig. 2D). This decrease in standard deviation is associated with a decrease in coefficient-of-variation for the total PN conductance. Qualitatively speaking, the PN is more ‘mean driven’ when 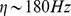, and the PN is more ‘fluctuation driven’ when 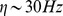, even though the firing-rate of the PN is similar in both cases (Fig. 2E and Fig. 2F). This can be quantified by measuring, for example, the autocorrelation of the PN. In the case 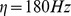, the PN autocorrelation shows several significant peaks, the first of which is at 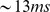, indicating periodic-firing at 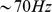 (Fig. 2E). In the case 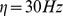, the PN autocorrelation does not indicate a strong periodicity to the PN firing-patterns (Fig. 2E).

The simple cartoon described above only considers synaptic-depression resulting from vesicle-depletion. The real AL displays evidence of presynaptic-inhibition as well. Nevertheless, the same general principle still holds regardless of the source of synaptic-depression at the ORN synapses, as long as the PNs become more mean driven as ORN firing-rates increase. In fact, it is possible to show analytically that similar results hold across a wide range of parameters for an idealized system similar to this one (see the section entitled “A simple analyzable cartoon of variance coding” in Methods).

If this picture is accurate in the real AL, then the PN dynamics within any given glomerulus in the AL will change as a function of ORN input to that glomerulus, even when the mean PN firing-rates have saturated for that glomerulus. These dynamical changes will only be observable through measurements of statistics that are ‘higher-order’ than mean firing-rate. We note that synaptic-depression of the ORN synapses is not the only mechanism via which the PNs may become more mean-driven as ORN firing-rates increase — other mechanisms, such as spike-frequency adaptation, could also contribute to this effect. As long as the postsynaptic influence of each ORN spike decreases as 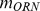 increases, the PN activity will become more mean-driven as 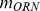 increases. As the PN activity becomes more mean-driven, we expect the firing-sequences produced by that PN to become more regular [22].

#### An illustration of variance coding within a large-scale model

We also observe this phenomenon within our large-scale model (described in Methods), which contains both presynaptic-inhibition and vesicle-depletion. To illustrate this phenomenon at work, we created a panel of 16 odors, all of which saturated the PN firing-rates (i.e., produced average PN firing-rates at the ‘plateau’ of the 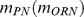 curve for the model). We presented each of these odors to the model network 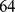 times.

For each of the 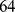 trials of each stimulus we measured the 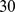-component vector of PN firing-counts collected over the 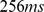 following odor onset. Each component of this vector represents the number of spikes fired by one of the 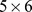 PNs during this time. We then used this vector to perform each possible 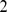-way and 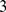-way stimulus discrimination task (see the section entitled “Odor Discrimination” in the Methods). Each of these 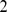-way and 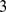-way discrimination tasks results in a discriminability rate (i.e., the fraction of correctly categorized trials – note that chance performance for a 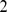-way task is 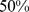, and chance performance for a 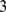-way task is 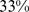).

**Figure 3:**
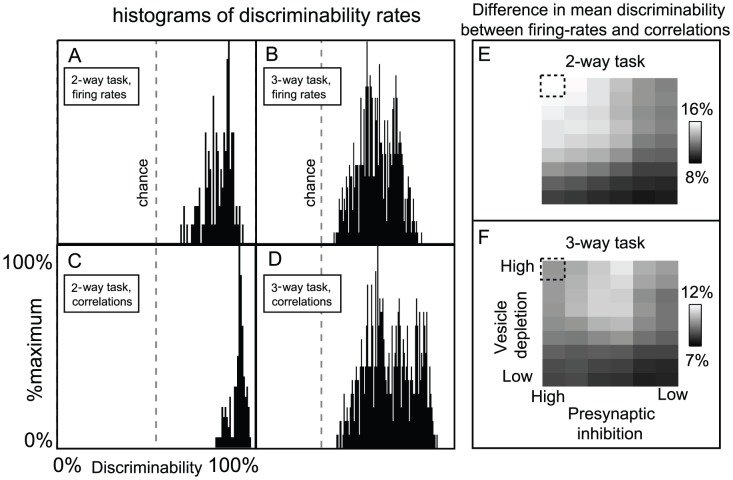
A manifestation of variance coding within the large-scale model. The large scale model (described in Methods) exhibits a phenomenon similar to the variance coding shown in Fig. 2. We constructed a panel of 16 odors, all of which only directly stimulated the same 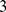 glomeruli (although to differing degrees). Moreover, we chose every odor within this panel such that the ORN firing-rates of the 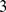 directly stimulated glomeruli were sufficient to saturate the firing-rates of the associated PNs (i.e., the directly stimulated ORN firing-rates were 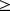
12 Hz, see Fig. 10). Given this panel of odors, we presented each odor multiple times, and used the collection of 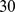-component PN firing-rate vectors (measured over the 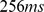 period immediately following odor onset) to perform a variety of odor discrimination tasks (see Results for details). [A] The histogram of discriminability rates associated with 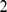-way discrimination tasks when only firing-rate data is used. Note that 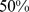 is chance level for these tasks (chance level is also shown in panels B,C,D). [B] The histogram of discriminability rates associated with the 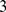-way discrimination tasks when only firing-rate data is used (note that 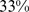 is chance level for these tasks). [C] The histogram of discriminability rates associated with 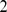-way discrimination tasks when firing-rate data and 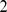-point correlations (correlation time 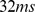) are used. [D] The histogram of discriminability rates associated with 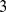-way discrimination tasks when firing-rate data and 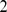-point correlations (correlation time 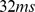) are used. Note that the typical discriminability rate is higher when correlations are used. [E] Here we plot the difference in mean discriminability for the 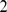-way discrimination task between the cases (i) when firing-rate data and 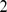-point correlations are used, and (ii) only firing-rate data is used. We plot this difference as a function of the parameters 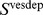 and 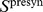 used in our large-scale model. The vesicle-depletion parameter 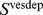 ranges from 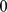 to 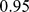 across the vertical axis, and the presynaptic-inhibition parameter 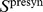 ranges from 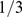 to 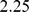 across the horizontal axis. The data shown in panels A–D is taken from the simulation indicated by the dashed square. Note that, as the total amount of synaptic-depression decreases, the discriminability computed using only firing-rates is closer to the discriminability computed using both firing-rates and 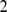-point correlations. [F] Similar to panel-E, except for the 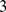-way discrimination task, rather than the 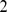-way discrimination task.

We construct a histogram of the discriminability rates for the 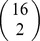 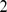-way discrimination tasks, and as expected (see Fig. 3A), the typical discriminability rate for the system is not particularly high (recall that each odor saturated the PN firing-rates). Similarly, the 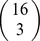 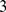-way discrimination tasks performed using PN firing-rate vectors also do not yield high discriminability rates (Fig. 3B). However, if instead of merely using PN firing-rate information we also use information regarding PN-PN correlations within the system, then the typical discriminability rates for the 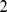-way and 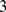-way tasks increase (see Fig. 3C,D). To produce the discriminability rates shown in Fig. 3C,D, we measured not only the 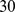-component vector of PN firing-counts for each odor trial, but also the 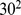-component vector of PN-PN correlations (with correlation time 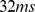). As expected, these higher-order statistics contain enough information to discriminate odors significantly more reliably than mere firing-rates.

The difference between the performance of these low-order and high-order readouts is more noticeable when the synaptic-depression in the system is strong. Conversely, in a network with no vesicle-depletion and reduced presynaptic-inhibition, the low- and high-order readouts yield more similar discriminability-rates (see Fig. 3E,F). Thus, the presence of strong synaptic-depression within our system is one factor which allows the network's dynamics to encode input-specific information within the PN-PN correlations.

For the example shown in Fig. 3, the difference between the typical 2-way discriminability rates observed when using high-order versus low-order readouts is maximized when the synaptic-depression is strongest; the effect of variance coding is seen quite clearly. However, for the 3-way discriminability rates, the difference between the high- and low-order readouts is greatest when the presynaptic-inhibition is not too strong. A natural question is: why does the performance for the 3-way discrimination task not parallel that for the 2-way task? Why is the difference in performance between high- and low-order readouts not maximized when both presynaptic-inhibition and vesicle-depletion are at their strongest?

This effect arises in part because the 3-way task is quite difficult and the observation time 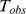 over which the task is carried out is rather short — 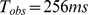 in this case. As we will argue below, one consequence of strong presynaptic-inhibition is that the network's ability to perform fine discrimination will be compromised when 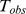 is small. In order to perform very well on fine discrimination tasks when 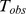 is small, the network should have only moderate amounts of presynaptic-inhibition (consistent with Fig. 3F).

### Hypothesis 2: a tradeoff between reliability and sensitivity

It has been hypothesized that one functional role for the AL is to separate similar odors and that the nonlinear gain curve 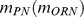 is instrumental in this process. As shown in [11], the nonlinearity of 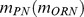 is influenced strongly by substantial synaptic-depression at the ORN synapses. Thus, it is reasonable to conclude that one functional role of synaptic-depression at the ORN synapses is to enhance the odor separation capabilities of the AL.

Within the fly AL there are multiple sources of synaptic-depression at the ORN synapses. Two major mechanisms which contribute to this synaptic-depression are vesicle-depletion and presynaptic-inhibition. While either one of these mechanisms could, in principle, be the major contributing factor to the synaptic-depression observed within the fly AL, it seems as though both of these mechanisms play a substantial role in producing synaptic-depression [11], [12]. Thus, one is faced with the following natural question: What purpose do these two distinct mechanisms serve within the fly AL? How would the odor-coding properties of the fly AL change if, say, only one of these mechanisms were responsible for the observed levels of synaptic-depression at the ORN synapses? Is there some functional advantage gained by having both of these mechanisms at play?

In what follows we introduce a hypothesis which links the underlying nature of synaptic-depression at the ORN synapses to information-coding properties of the AL, such as reliability, sensitivity and discriminability. First we will define these terms, and then we will explain our hypothesis in more detail throughout the rest of this section.

**sources of noise**: There are two sources of ‘noise’ in our network which influence the reliability (or unreliability) of the AL's activity across trials. The first is the initial condition of the system (i.e., the state of the system at odor onset). Different initial conditions will give rise to different dynamic trajectories. The second source of noise is the odor-driven Poisson input to the ORNs in the model. Different trials will give rise to different sequences of ORN spikes.

**reliability**: We define the reliability of the AL as the inverse of the coefficient-of-variation in spike-counts of AL neurons, as measured across trials over a given stimulus-driven time-window. Reliability is high if the spike-counts of the AL neurons are similar from trial-to-trial. Reliability is low if the spike-counts vary significantly from trial to trial. In our analysis we will consider a family of networks with the same mean firing-rate, hence the notion of reliability can be constructed using standard-deviation in spike-counts across trials, rather than coefficient-of-variation.

**sensitivity**: Given two similar stimuli, we can measure the time-averaged firing-rates of the various neurons in the AL, collected over a long time (e.g., 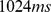). If the firing-rates induced by these two similar stimuli are nearly identical, we say that the AL is ‘not sensitive’ to the difference between these two stimuli. On the other hand, if the firing-rates induced by these two stimuli are quite different, then we would describe the AL as ‘sensitive’ to the stimulus difference. More specifically, we define sensitivity to be the magnitude of the derivative of the vector of steady-state AL-firing-rates, when considered as a function of the odor input. In this sense, our notion of sensitivity is built around firing-rates, and does not explicitly consider higher order dynamical structure.

**discriminability**: Given an unknown odor from amongst a set of possible known candidates, we can use the AL as a discriminator: by presenting this mystery odor to the AL and measuring PN firing-counts over a time-period 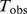, we can attempt to classify the input as one of the possible candidate odors. We define the discriminability of the AL as the accuracy (i.e., correct-classification rate) of this procedure. The discriminability depends strongly on 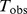. If 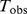 is sufficiently long, the discriminability of the AL is related directly to its sensitivity. If 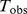 is short, then unreliability may come into play and reduce discriminability. As with our definition of sensitivity, our definition of discriminability is built around measurements of firing-rates, and does not take into account higher order dynamic structure.

The main thrust of our second hypothesis is that the combination of the mechanisms of vesicle-depletion and presynaptic-inhibition allows the fly AL to balance sensitivity and reliability in such a manner as to maximize the discriminability of AL activity (with respect to similar ORN inputs) over short observation times.

#### An illustration of the tradeoff between reliability and sensitivity within a large-scale model

In this subsection we will show how the hypothesis introduced above manifests within our large scale model. First we will discuss some features of this model which are pertinent to this hypothesis, then we will discuss our hypothesis in more detail.

We used simulations to investigate and benchmark our large-scale model (see the sections regardin benchmarking in the Methods). By analyzing these simulations we determined that, even after benchmarking, there were still a handful of free parameters that were left unconstrained. Two parameters in particular were not fully constrained by our benchmarking: (i) the strength of vesicle-depletion as characterized by 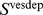, and (ii) the strength of presynaptic-inhibition as characterized by 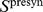. Within our large-scale model the combination of these two parameters produced synaptic-depression of the ORN synapses. While the total amount of synaptic-depression was constrained by our benchmarking, the relative strengths of 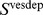 versus 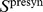 were not constrained.

As an example of this lack of constraint, consider the following benchmark: assume that we expect the average PN firing-rate within the AL to saturate at a certain level 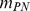 when stimulated sufficiently by ORN input. What we found was that there is a spectrum of possible AL architectures which could produce this desired firing-rate 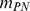: (A) on one end of the spectrum is an AL in which there is hardly any vesicle-depletion of the ORN synapses, but for which the LNIs give rise to substantial presynaptic-inhibition at these synapses. This type-A AL would be characterized by a large value of 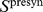 and a small value of 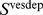. (B) on the other end of the spectrum is an AL in which vesicle-depletion is primarily responsible for synaptic-depression, and the presynaptic-inhibition of the ORN synapses due to LNIs is negligible. For this type-B AL 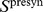 would be small and 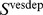 would be large.

**Figure 4:**
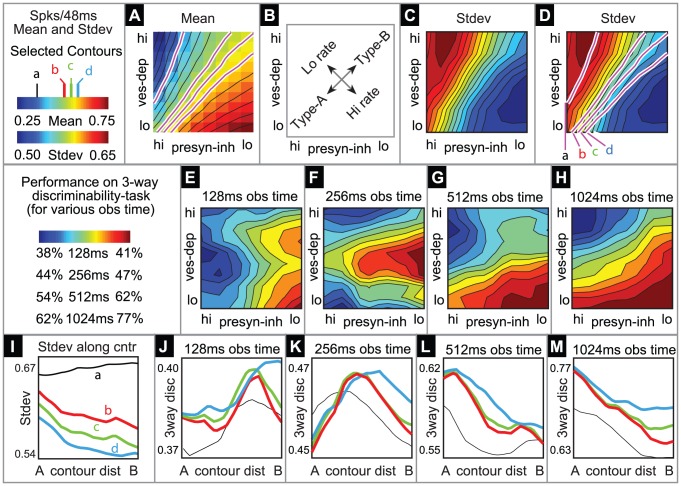
A tradeoff between reliability and sensitivity within our large-scale model. We performed a systematic scan of our large-scale network model, varying 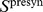 and 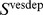 (see the section entitled “An illustration of the tradeoff between reliability and sensitivity within a large-scale model” in the main text for details). For each point in this parameter array we measured various features of the network dynamics (such as mean PN spike-counts and reliability), as well as the performance of each of these networks on a 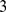-way odor discrimination task. [A] Shown is the mean PN spike-count of PNs in the first glomerulus, for each pair of parameter-values 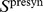, 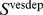. Overlaid on top of the mean spike-counts are contour lines for the spike-count. Four of these contours are highlighted in magenta, and will be referenced later. [B] Indications of the type-A and type-B network regimes. [C] Shown are the standard deviation in PN spike-counts of PNs in the first glomerulus (see colorbar on far left). [D] Reproduction of panel-C, along with the contours of panel-A. [E–H] Shown are contour plots associated with 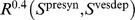 for various values of 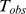. These panels use the colorbar shown to the far left. [I] Here we plot the standard-deviation in spike-count (taken from panel-D) as a function of the distance along each of the contours indicated in panel-D, with values bi-linearly interpolated as necessary. [J] Here we plot the discriminability values 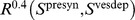 indicated in panel-E as a function of the distance along each of the contours shown in panel-D. The contours are indicated using the colorcode from panel-I. [K–M] Similar to panel-J, except for 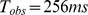, 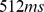, and 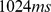 respectively.

An example of this spectrum is given in Fig. 4. Given a fixed value 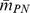 for the saturated firing-rates of PNs in a strongly driven glomerulus, there exists a 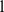-parameter family of values 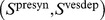 which corresponds to networks exhibiting saturated firing-rates equal to 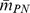. This 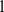-parameter family of values ranges from networks with high 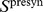 and low 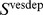 (i.e., type-A networks) to networks with high 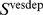 and low 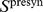 (i.e., type-B networks).

Shown in Fig. 4A are the mean stimulus-driven PN spike-counts for several networks with varying values of 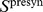 and 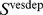. To construct this example we performed a systematic scan of parameter space for our large-scale network model. We selected a 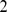-dimensional array of parameter values for 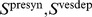, ranging from 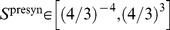 and from 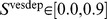. For each fixed 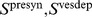 within this array, we ran a large-scale simulation using a panel of 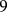 odors, and we ran 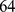 trials per odor. The first eight of the odors used stimulated three glomerular channels — the first glomerular channel was stimulated strongly, and an odor-specific subset of two other glomerular channels was stimulated weakly. The ninth odor only stimulated the first glomerular channel strongly. We remark that the simulations used to construct this array differ only in their values of 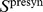 and 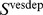. The architecture and connectivity of the rest of the model network were fixed.

In Fig. 4A we show the mean spike-count of PNs in the first glomerulus, for each pair of parameter-values 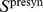, 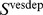. The mean spike-count is calculated as the mean of the number of spikes/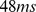 time-bin averaged across all 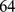 trials, and further averaged over the 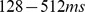 period following odor onset, and further averaged across all 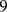 odors. Overlaid on top of the mean spike-counts are contour lines for the spike-count. Each of these contours represents a 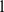-parameter family of networks with a different constant mean stimulus-induced spike-count. Note that, as indicated in Fig. 4B, these contours extend from regions of high 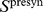 and low 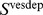 to regions of low 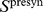 and high 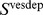. In this example Type-A networks correspond to the lower-left corner of the array, and Type-B networks correspond to the upper-right corner of the array. Thus, in Fig. 4A it can be seen that 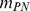 is constant along contours extending from type-A networks (lower left) to type-B networks (upper right).

We observed two important systematic differences between the candidate networks along these 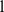-parameter families. First, type-B networks are more reliable than type-A networks. This can be understood as follows. First consider the ORN inputs to PNs in a type-B network (for which synaptic-depression is dominated by vesicle-depletion). A typical odor stimulates many ORNs to fire at a high rate. Each of the ORN synapses likely has a high quantal release rate [11], implying that the fraction of active vesicles remaining after several rapid ORN spikes is likely to be small. Moreover, there are 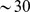 such ORNs which converge onto each PN within their target glomerulus [23]. Thus each PN within a strongly stimulated glomerulus receives a large number of input spikes from a large number of presynaptic ORNs, each firing with a high rate, each synapse of which is likely to experience profound vesicle-depletion. Moreover, the vesicle-depletion experienced by the ORN synapses is only dependent on the ORN activity, and is independent of the activity of the AL. Thus, we expect the ‘feed-forward’ synaptic-depression observed within a type-B network to always exhibit very similar dynamic transients from trial to trial, with the only differences due to the variation in ORN spike-sequences induced by the trial-to-trial variability of the Poisson input to the ORNs [19]. Now, on the other hand, let us consider the ORN inputs to PNs within a type-A network. In such a network, synaptic-depression is primarily governed by ‘feedback’ from the AL in the form of presynaptic-inhibition. ORNs in a type-A network rely on the odor-specific firing patterns of LNIs in order to exhibit synaptic-depression, and therefore may receive different amounts of presynaptic-inhibition from trial to trial (or over disjoint time-windows within a single trial). Moreover, there are only a few LNIs per glomerulus, and a given stimulus may not cause all these LNIs to fire at high rates. A few extra LNI spikes induced on any one trial may substantially change the footprint of synaptic-depression across the ORN synapses, thus leading to even more extra LNI spikes later on, and so forth. This ‘feed-back’ mechanism allows the synaptic-depression observed within type-A networks to exhibit quite different dynamic transients from trial to trial. Put another way, the ‘feed-back’ structure within type-A networks allows the trial-to-trial variability in LNI activity to affect and magnify the trial-to-trial variability in ORN input to the AL. In conclusion, we expect that ORN inputs to PNs in type-A networks will be less reliable than the corresponding ORN input to PNs for type-B networks when measured either (a) over multiple trials, or (b) over different time-windows within a single trial.

The second systematic difference between networks along such a 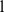-parameter family is that type-A network-dynamics is more sensitive than type-B network-dynamics to subtle changes in ORN input. To see why this might be true, let's revisit the argument used above. Consider a subtle change in ORN input which is only large enough at first to shift PN and LN firing rates slightly. This subtle change in ORN input will not create a large shift in the PN input for type-B networks, yet the same subtle change in ORN input may give rise to a few different LNI firing-events in the type-A network, which may then presynaptically inhibit different ORNs, giving rise to even more different type-A-network-activity, and so forth. In other words, due to the feedback between the type-A LNIs and the type-A ORNs, we expect the type-A system's dynamics to be more sensitive than the type-B network's dynamics to certain perturbations in input.

These systematic differences (i.e., type-A networks are less reliable, but more sensitive to perturbations in input than type-B networks) manifest within our large-scale model.

To quantify reliability for each network along such a 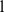-parameter family, we measured the trial-to-trial standard deviation in PN spike-counts of PNs in the first glomerulus, for each pair of parameter values 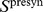, 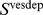. The standard-deviation is calculated as the standard-deviation of the number of spikes/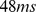 time-bin across all 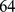 trials, averaged over the 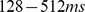 period following odor onset, then further averaged across all odors. The coefficient-of-variation in spike-counts is equal to the standard-deviation in spike-count divided by the mean. Thus, along contours of 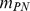 (where the mean is constant) the coefficient-of-variation in spike-count will be proportional to the standard-deviation in spike-count. Shown in Fig. 4D are the standard deviation in PN spike-counts along with the 4 contours highlighted in Fig. 4A. These four contours (labelled 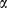,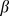,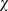,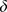) each correspond to a 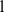-parameter family of networks exhibiting a fixed mean spike-count, and are each associated with a different color (black, red, green, cyan, respectively) on the colorbar to the far left. In Fig. 4I we plot the standard-deviation evaluated along these contours. Note that, since contour 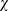 is longer than contour 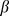, the graphs shown in Fig. 4I are not directly comparable. However, there is a clear trend amongst all these graphs: As one moves along the 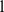-parameter family of networks with constant mean stimulus-induced spike-count from type-A networks to type-B networks the standard-deviation in spike-count decreases as long as the mean spike-count is sufficiently high (i.e., contours 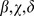). This is equivalent to the statement that, along contours 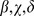, type-B networks are more reliable than type-A networks.

Recall that, for each network (i.e., for each fixed value of 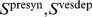), we ran 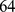 trials for each of 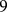 different odors. Using this data, we can quantify the sensitivity of each of these networks to input perturbations. For each odor trial we measure the 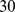-component PN firing-rate vector averaged over the 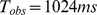 time-window including and immediately following a 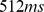 odor presentation. We use these time-averaged firing-rate vectors to perform each of the 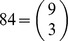 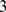-way odor discrimination tasks, and thus we obtained a distribution of discriminability rates for each 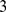-way odor task (see the section entitled “Odor Discrimination” in the Methods). For each network we then record the 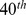-percentile of the distribution of discriminability rates (across odors), denoted by 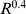. We chose to display 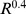, as this 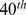-percentile discriminability rate illustrates our conclusions most clearly. However, our main results do not change if we choose another percentile in the range 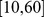. Higher percentiles, such as the 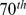-percentile, are usually all near 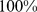 correct-classification, since the set of odors used contain several rather distinct odors. Note that 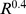 will depend on 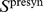 and 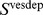. Shown in Fig. 4H are the contour plots associated with 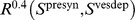 for 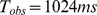. In Fig. 4M we plot these discriminability rates along the contours shown in panel-A. For each of these contours the maximum discriminability (when 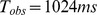) occurs at the type-A end of the spectrum. This indicates that the discriminability of type-A networks (using firing-rates measured over long observation times) is superior to that of the type-B networks. This is a reflection of the fact that type-A networks are more sensitive than type-B networks to subtle changes in input.

#### A combination of vesicle-depletion and presynaptic-inhibition is required to optimize discriminability over short observation-times within a large-scale model

Within our model network we have observed a further functional consequence associated with the tradeoff between reliability and sensitivity described above. Type-A networks are indeed more sensitive than type-B networks to shifts in input, and this sensitivity is reflected in the long time (or trial averaged) PN firing-rate vector associated with any given input. As a result, type-A networks outperform type-B networks in odor-discrimination tasks when the discriminability rate is calculated using a long time observation (such as 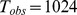, as shown in Fig. 4H,M). However, type-A networks are less reliable than type-B networks, and thus, if the observation-time of any given odor stimulus is sufficiently short, the higher variability associated with the single-trial short-time responses of type-A networks will pollute the performance of any discrimination task which uses only these short observations. On the other hand, since type-B networks are rather reliable, shortening the observation-time associated with a discrimination task will not affect the discriminability rate associated with that task for a type-B network as much. As demonstrated in our model network, if the observation-time of any given odor trial is shortened from 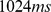 (as shown in Fig. 4H,M) to merely 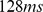 after odor onset, the decreased reliability associated with type-A networks will drastically lower the discriminability rate of the odor-discrimination tasks which use only these short observations (see Fig. 4E,J). Moreover, since the type-B networks are more reliable than type-A networks, the decrease in discriminability associated with reducing the observation-time of the discriminability task is lower for type-B networks than it is for type-A networks (compare Fig. 4J,M). Most intriguingly, there is a midpoint in the spectrum — a balance between vesicle-depletion and presynaptic-inhibition — which gives rise to the maximum discriminability rates using only short-time observations. This optimal point depends on the length of the observation-time associated with the discrimination-task. With long observation-times type-A networks are optimal. With very short observation-times type-B networks are optimal.

This feature is shown in more detail for our large scale model in Fig. 4E,F,G,J,K,L, which illustrate the discriminability capabilities of our model for a variety of observation times 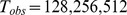. Note that, for any particular contour 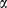,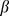,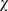,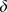, The point of maximum performance occurs closer to the type-B extreme when 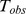 is small, and this maximum occurs closer to the type-A extreme when 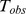 is large. In other words, when 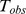 is low, type-B networks outperform type-A networks, whereas when 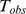 is large type-A networks outperform type-B networks.

In conclusion, we have demonstrated that for a particular set of discrimination tasks the network which performs optimally lies in between the type-A and type-B extremes. Moreover, as the observation-time associated with this task increases (or decreases) the optimal point shifts towards the type-A (or, respectively, type-B) end of the spectrum. Although the details of Fig. 4 only pertain to a particular discrimination task, we mention now that this systematic dependence of the optimal point on observation-time is actually a natural consequence of the fact that type-A networks are more sensitive, and type-B networks are more reliable. Indeed, as we will argue below (in a section entitled “A simple cartoon of optimizing discriminability over short observation-times”), this feature is to be expected for a rather general class of discrimination tasks in which estimates of the mean firing-rates (sampled over an observation-time) are used to classify the input.

#### A simple analyzable cartoon of the tradeoff between reliability and sensitivity

In this section we introduce a simple deterministic 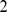-neuron model network which will allow us to discuss various aspects of hypothesis-2. This simple network has the property that the sequence of neuronal firing-events is a sensitive function of the network's initial conditions as well as the input to the network and the source of synaptic-depression within the network. The model itself consists of 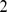 LNIs, each driven by a single ORN. Each LNI (labelled 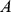 and 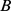) is modeled by a simple phase-oscillator (similar to a current-based integrate-and-fire neuron), and each ORN is modeled by a fixed input-current (i.e., 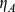 and 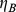). This input current indicates the rate at which each neuron would fire if there were no presynpatic-inhibition or vesicle-depletion.

In this system the strength of presynaptic-inhibition is modeled by a constant parameter 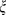. As 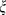 increases, the firing-events of each neuron have a greater inhibitory effect on the input to the other neuron. Specificially, whenever LNI 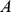 fires, the ORN input to LNI 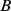 is shut off for 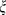-time. Similarly, whenever LNI 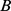 fires, the ORN input to LNI 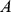 is shut off for 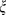-time. To ensure that both neurons fire, we assume 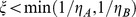. As the amplitudes 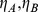 of the ORN processes are constant, the vesicle-depletion in this system is assumed to attain a steady state, and is modeled via a single constant parameter 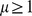, which reduces the ORN input to both 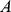 and 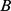.

In keeping with the description above, the membrane potentials for LNI 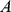 and 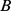 obey the differential equations
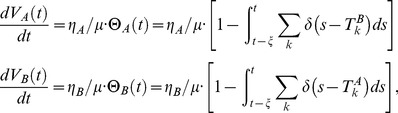
(1)and whenever the potential 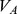 reaches 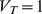, we say that LNI 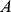 fires, and reset 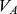 to 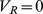. The 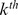 spiketime of neuron 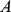 is recorded as 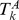. Similarly, whenever 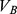 reaches 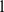, we say that LNI 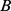 fires, and record the 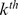 spiketime of neuron 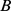 as 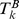. The term 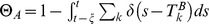 is equal to 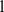, unless neuron 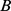 has fired within 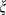 of the current time, in which case 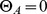. Similarly, the term 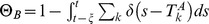 is equal to 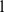, unless 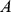 has fired within 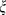 of the current time, in which case 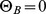. Note that, since 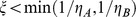, the terms 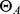 and 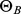 are each either 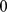 or 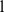 at each time.

This simple network is easy to analyze, and the firing-rates 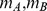 of each LNI in the network, as well as the interspike-interval-distributions (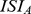, and 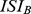) can be directly calculated in terms of the inputs to the network 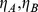 and the sources of synaptic-depression 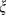, and 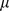. See the section entitled “A simple model illustrating the tradeoff between reliability and sensitivity” in the Methods for more details.

**Figure 5:**
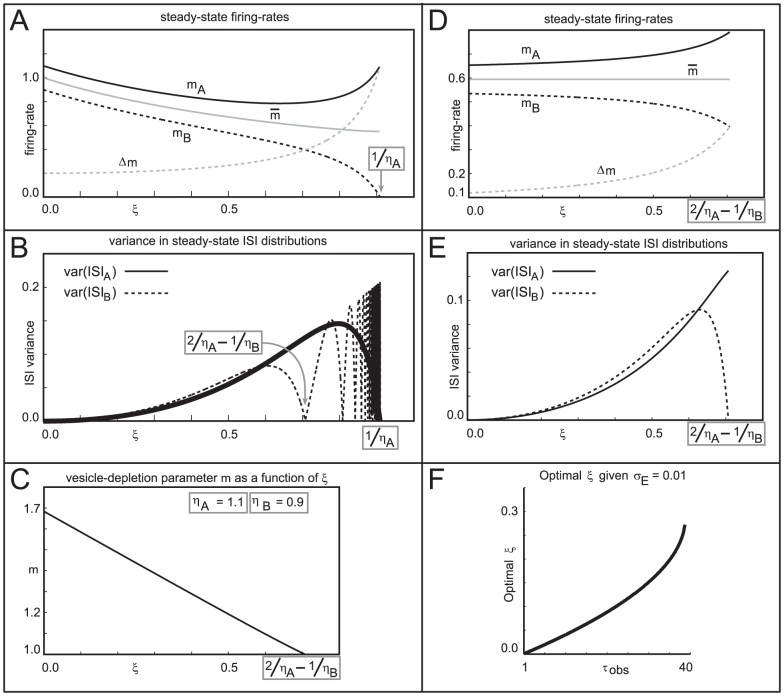
A simple analyzable cartoon of the tradeoff between reliability and sensitivity. In this example 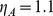, and 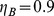. In panels A and B the vesicle-depletion parameter 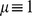. In panels C,D,E and F, the vesicle-depletion parameter 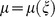, such that the mean firing rate 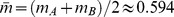 is held constant. [A] Graphs of 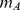 (solid), 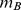 (dashed), 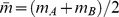 (gray), and 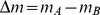 (gray dashed), as functions of 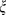, for the case 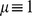. [B] Graphs of var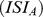 (solid) and var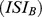 (dashed) as functions of 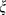, for the case 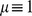. [C] Graph of 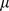 as a function of 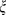, subject to the constraint that 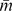 remain constant. The constant value of 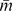 chosen (essentially arbitrarily) in this case is the value of 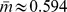 shown in panel A for 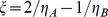. Other choices of 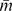 yield similar results. Note that this graph is monotonically decreasing, implying the existence of a 1-parameter family of networks possessing the same 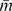 — ranging from type-A networks with low 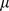 and high 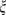, to type-B networks with high 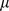 and low 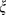. [D] Graphs of 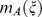 (solid), 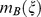 (dashed), 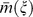 (gray), and 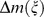 (gray dashed), for the case 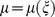. [E] Graphs of var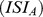 (solid) and var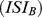 (dashed) as functions of 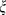, for the case 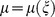. [F] Graph of the optimal choice of 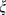 (implying a vesicle-depletion parameter of 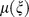) for which discriminability is maximized, as a function of the sample number 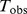. The notion of discriminability is described in the section entitled “A simple cartoon of optimizing discriminability over short observation-times”. In this case the observation error 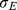 is fixed at 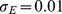. Note that for low 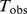, discriminability is maximized for a type-B network. However, as 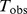 increases, discriminability is maximized by type-A networks. The graph shown plots 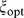 for 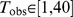, as for this particular simple example the derivative of 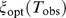 reaches a vertical asymptote at 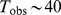.

An example of such calculations is shown in Fig. 5. If we fix 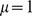, then the firing-rates of the two neurons is a decreasing function of 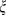 for small 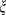 (see Fig. 5A). As expected, this decrease in firing-rate corresponds to the two neurons interfering with and slowing down one another. However, this interference causes the firing-events of each neuron to occur at irregular intervals, and hence the variance in the ISI-distributions of these neurons is a monotonic *increasing* function of 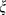 for small 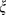 (see Fig. 5B). Thus, as 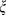 increases from 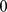, the neurons fire less, and have a lower trial-to-trial reliability.

If we fix 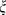, then the firing-rates of the two neurons are decreasing functions of 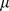. Thus, there is clearly a 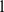-dimensional family of synaptic-depression parameters which gives rise to networks exhibiting the same mean firing-rates for any fixed set of inputs. This 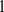-parameter family ranges from type-A networks (with high 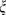 and low 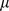) to type-B networks (with low 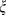 and high 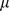) – see Fig. 5C. We can index networks along this 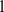-parameter family using 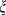, assuming that 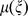 is chosen so that the average firing-rate 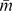 is maintained (see Fig. 5D).

Intuitively, one expects that for an extreme type-B network (i.e., 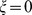) the activity should be perfectly regular: each neuron fires independently of the other neuron. On the other hand, for an extreme type-A network (i.e., 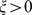), each neuron fires in spurts, constantly disrupting the periodicity of the other neuron's activity. This interplay between the neurons (resulting from presynaptic-inhibition) gives rise to a greater variability in the ISI-distributions of the neurons within the type-A networks. This increased variability implies that the neurons in the type-A networks have a lower trial-to-trial reliability than the analogous neurons within the type-B networks (assuming that different trials have different initial conditions).

This same intuition can be extended to see that, in the type-A network, there is a ‘rich-get-richer’ phenomenon: the neuron which gets more input will slow the other neuron down more than it is slowed down by the other neuron. Thus, the presynaptic-inhibition 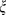 magnifies the sensitivity of the type-A networks, increasing the difference in firing-rates between the two neurons when the input to these neurons is similar. In other words, the firing-rates produced by the type-A network should be more sensitive than those produced by the type-B network to small differences in inputs.

This intuition is borne out by analysis. When constraining 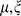 so that the mean firing-rate 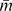 is constant, we see that as 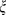 increases from 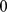 the network becomes more sensitive (i.e., the difference in firing-rates 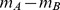 increases) and less reliable (i.e., the variance of the ISI-distributions of the two neurons increases). These functions are plotted in Fig. 5D,E. Thus, for this simple system, we can show analytically that hypothesis-2 holds: type-A networks are more sensitive, and type-B networks are more reliable. See the section entitled “A simple model illustrating the tradeoff between reliability and sensitivity” in Methods for more details.

#### A simple cartoon of optimizing discriminability over short observation-times

As postulated above, and illustrated for both a large-scale and idealized network architecture, we expect there to be a 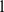-parameter family of networks with the same mean firing-rate for any fixed set of inputs. This 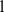-parameter family ranges from type-A networks (with significant presynaptic-inhibition, lower reliability and higher sensitivity) to type-B networks (with significant vesicle-depletion, higher reliability and lower sensitivity). It turns out that, under rather general conditions, the networks which perform best on discriminability tasks with finite observation-times are the networks in the middle of this spectrum (i.e., networks with a combination of presynaptic-inhibition and vesicle-depletion).

To illustrate this principle, we will use the network architecture discussed in the section entitled “A simple analyzable cartoon of the tradeoff between reliability and sensitivity”. It should be noted however, that the argument we will present here is not specific to the 2-neuron architecture discussed above, and a modified version of this argument will hold for any 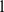-parameter family of networks ranging from the type-A to the type-B extremes discussed above.

To begin, let us consider the following discriminability task. Assume that a simple 2-neuron network (of the type described in the section entitled “A simple analyzable cartoon of the tradeoff between reliability and sensitivity”) is driven by one of two inputs — either (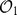) neuron 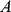 is driven at rate 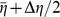 and neuron 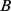 is driven at rate 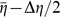, or (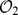) 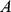 is driven at 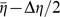 and 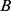 is driven at 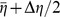. The steady-state ISI distributions 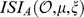 and 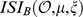 will depend on the unknown input 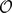, as well as the known system parameters 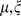. By performing a measurement of the system, is it possible to tell which input (i.e., either 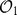 or 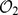) is driving the system? Let us assume that our measurement process consists of 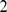 steps. First, we estimate the mean of 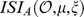 by drawing 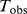 samples from 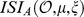. For example, we can either measure 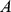 within a single system for a long time, or we can measure multiple systems from an ensemble. The longer a single system is measured for, the larger the effective number of samples 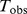 is, assuming that measurements of the system that are sufficiently well-separated in time are effectively independent. We use the notation 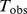 rather than 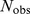 to draw analogy with the observation-time discussed in the section entitled “A combination of vesicle-depletion and presynaptic-inhibition is required to optimize discriminability over short observation-times within a large-scale model”. Let us denote by 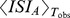 this estimate for the mean of 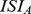. Second, we assume that our measurement process includes some external noise modeled by a random variable 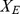. For the sake of presentation, let's assume that 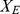 is drawn from 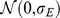 (i.e., a Gaussian distribution with mean 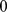 and variance 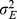). Thus, our final measurement of the mean of 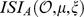 is some estimate 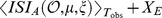.

Our goal is to determine from this measurement 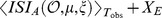 whether the input to the system is 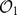 or 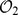. By analyzing the signal to noise ratio of this measurement process (see the section entitled “Analysis of signal-to-noise ratio in a general discrimination task” in Methhods), we can show that the discrimination error 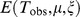 associated with the best linear-classifier for this problem is well-approximated by:
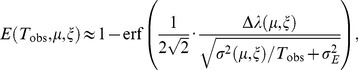
(2)where 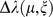 is the difference in the means of 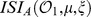 and 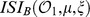, and 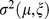 is the average variance of 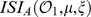 and 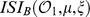. Because 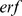 is monotonic increasing, 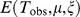 is minimized when the ratio 
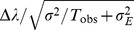
 is maximized. Recall the structure of the simple 2-neuron networks described above — both 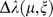 and 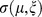 are monotonically increasing functions of 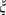, and 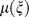 can be defined implicitly through 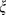 (by fixing 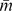) as a monotonically decreasing function of 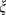. For these simple networks, when 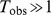, this discriminability error is minimized when 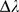 is as large as possible, and the maximum 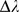 is achieved when 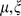 correspond to a type-A network. Conversely, when 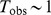, then the discriminability error is minimized for 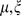 corresponding to a network in between the type-A and type-B extremes. In this case, one can show that if 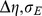 are sufficiently small, then the optimal 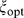 (for which the error 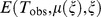 is minimized) increases as 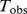 increases. (see Fig. 5F, which displays 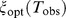 for the case 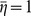, 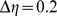, 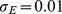).

As we mentioned earlier, The argument given in this section is quite general, and similar reasoning can be applied whenever any measurement is made by sampling from a distribution and adding an observation error 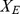. Given a 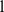-parameter family of networks indexed by 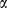, and a measurement of any dynamical feature 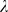, with sensitivity described by 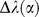 and reliability described by 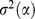, the error associated with the best linear-classifier can be approximated by an equation similar to Eq. 2, where 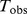 increases as the observation-time of the measurement increases.

#### A population-dynamics approach towards verifying Hypothesis 2 within more general networks

In the preceding sections we have described in detail a specific 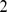-neuron network which exhibits the phenomena associated with hypothesis-2. However, the reasoning used in these sections cannot readily be applied to more complicated heterogeneous networks composed of more realistic model neurons. Indeed, while there exist networks for which hypothesis-2 holds (e.g., the large-scale networks described earlier on), there also exist networks for which hypothesis-2 does not hold. A natural question is: given a specific network architecture, what dynamic phenomena will that network exhibit? In the remainder of this section we will apply a rather general method [24], [25] which can be used to assess the equilibrium dynamics of pulse-coupled networks, and which can be used to determine which network architectures exhibit phenomena associated with hypothesis-2 (e.g., the tradeoff between reliability and sensitivity discussed above). With this analysis we will able to see that hypothesis-2 holds for a rather large class of networks, and in particular holds for a class of sparse randomly connected networks, provided that the network size is sufficiently large.

For the purposes of illustration, let us consider a network of 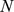 discrete-state glomeruli (LNIs), each driven by a different ORN. We will model each ORN-LNI pair as a discrete-state discrete-time Markov process which is as simple as possible, while still retaining the following features: (i) each LNI generates spikes, (ii) each ORN input spike contributes to the vesicle-depletion of that ORN
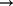
LNI synapse, and (iii), each LNI spike gives rise to presynaptic-inhibition of some subset of ORN
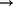
LNI synapses. This model does not take into account excitatory interactions; PNs and LNEs are not included. While these excitatory interactions certainly contribute to hypothesis-2, they do not substantially change the following analysis, and we delay discussion of their effects until the end of this section.

Within this simple network model we will model the 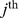 ORN-LNI pair using the state-variables 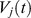, 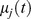 and 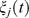 which represent LNI membrane-potential, ORN vesicle-depletion and ORN presynaptic-inhibition, respectively. The architecture of the model is determined by the connectivity matrix 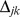 which encodes the presynaptic-inhibitory coupling between ORN-LNI pairs. The other parameters of the model include the feedforward input rates 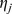 to each ORN-LNI pair, as well as the overall strength of vesicle-depletion 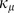 and the overall strength of presynaptic-inhibition 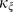. The details of the model are given in a section entitled “A discrete state model used to analyze hypothesis-2 within general networks with arbitrary architecture” in Methods (see Eq. 16).

We are interested in how the dynamics of such a network depends on the connectivity matrix 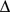, and also on other parameters such as the inputs 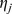, the vesicle-depletion strength 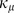 and the presynaptic-inhibition strength 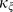. For reference, let us specify precisely what we mean by ‘hypothesis-2’. Let us say that the 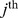 LNI satisfies ‘hypothesis 2.0’ if there exists a 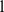-parameter family of small variations in 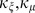 which maintain the firing rate of the 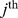 LNI (denoted by 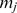), such that this 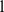-parameter family ranges from high 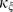 and low 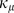 (i.e., type-A) to low 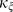 and high 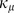 (i.e., type-B). Let us say that the 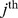 LNI satisfies ‘hypothesis-2.1’ if, given a small increase in 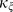 along this 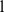-parameter family, the reliability of the 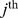 LNI decreases (i.e., var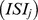 increases as the network parameters are shifted towards a type-A network). Finally, let us say that the 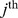 LNI satisfies ‘hypothesis-2.2’ if, given a small increase in 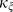 along this 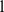-parameter family, the sensitivity of the 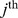 LNI to its own input increases (i.e., 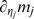 increases as the network parameters are shifted towards a type-A network).

Let us assume that we have some large network in which 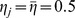 (i.e., the input to each LNI is the same), and that 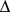 is given (but otherwise arbitrary). One can readily show that hypothesis-2.0 holds — namely, for sufficiently small 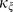, each ORN-LNI pair with at least one presynaptic-inhibitory input has the property that there exists a 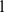-parameter family of parameters (ranging from high 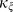 and low 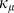 to low 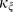 and high 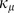) for which the firing rate 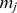 remains fixed. This is simply because, as either 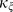 or 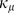 increases, the firing rate 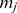 decreases (i.e., 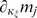 and 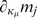 are both negative) as long as 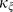 is sufficiently small.

In the rest of this section, we will analyze reliability (i.e., hypothesis-2.1). Let us concentrate on a single ORN-LNI pair (say, the 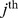 such pair) embedded within this larger network, and assume for the moment that the 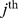 ORN is presynaptically-inhibited by the 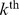 LNI (with 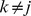). If we were to increase the strength of the presynaptic-inhibitory connection between LNI 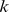 and ORN 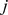 (i.e., if we were to increase 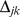) without decreasing the strength of vesicle-depletion 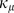, then the firing rate 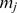 would drop. If, instead, we were to increase 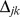 while decreasing 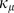 simultaneously so as to maintain 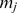 (as required by hypothesis-2.1), then the ISI distribution of the 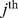 LNI would change (but the firing rate 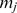 would remain constant by construction). In thi case the differential shift in the ISI distribution of the 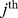 LNI associated with increasing the strength of connection 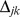 (while appropriately decreasing 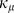) gives rise to an increase in var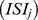. Thus, if coupling strengths are sufficiently weak, then the derivative of var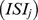 with respect to increasing 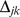 (while appropriately decreasing 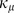) is positive. Using population-dynamics techniques from [24], [25], this reasoning can be systematically extended to consider every connection in the network, not merely the connection 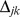.

**Figure 6:**
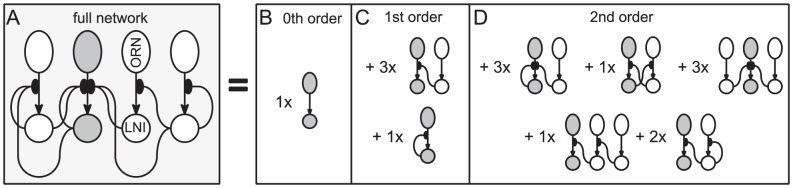
An example of subnetworks which come into play when considering the sensitivity or reliability of the 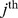 LNI. On the left in panel-A we show a particular network, with various ORN-LNI pairs (shown as ovals and circles respectively) connected via presynaptic-inhibitory connections. We will adopt the convention that the 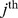 ORN-LNI pair is fixed (highlighted in dark gray), whereas the indices 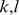 are not fixed, but are considered distinct from 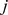 and from each other. Several dynamic features associated with the 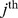 LNI can be determined by considering an expansion of the dynamics of this full network in terms of subnetworks. Shown on the right in panels-B,C,D are 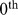-order, 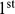-order and 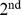-order subnetworks of the full network which are relevant for determining the sensitivity and reliability of the 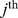 LNI. The 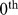-order subnetwork consists of the 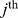 ORN-LNI pair alone. The two 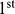-order subnetworks shown are those incorporating a single presynaptic-inhibitory connection — namely 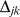 (top) and 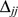 (bottom). The full network has embedded within it three 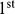-order subnetworks of the form 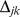, and one 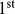-order subnetwork of the form 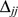. The five 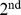-order subnetworks shown are those incorporating two presynaptic-inhibitory connections. Listed in reading order, these subnetworks are denoted by 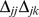, 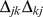, 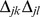, 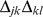, and 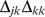. The full network has embedded within it 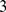, 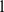, 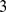, 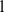 and 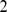 of these subnetworks, respectively.

**Figure 7:**
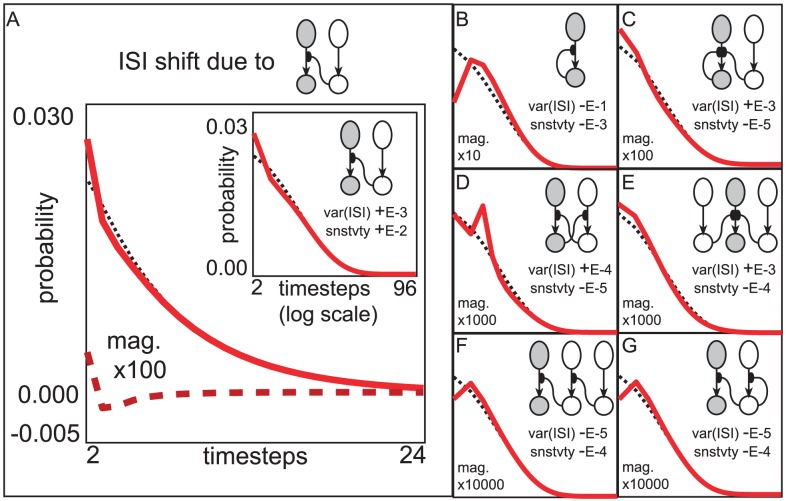
An example of subnetworks which influence reliability. In this example we assume a network of the form explained in the section entitled “A population-dynamics approach towards verifying Hypothesis 2 within more general networks”. The input to each LNI 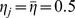 is constant, and the strength of vesicle-depletion 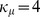. Note however, that we do not assume that the connectivity 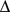 is fixed. We adopt the convention that 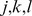 are distinct indices. [A] Here we illustrate the shift in the ISI-distribution of the 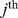 LNI (i.e., 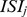) that would occur (up to 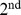-order) if the connectivity 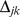 were increased while decreasing 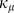 so as to maintain the firing-rate of the 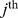 LNI (denoted by 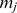). The ISI-distribution of the 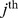 LNI when uncoupled from the rest of the network is shown with a dotted-line for reference. The rate at which 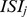 changes with respect to an infinitesimal increase in the coupling strength 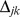 is shown with a dashed-line. This rate is magnified by a factor of 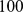 for visibility. The sum of this rate and the uncoupled 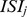 is shown with a solid-line for a qualitative representation of the new 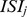 that would occur if the connectivity 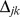 were increased by 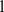. The inset shows this same data (dotted and solid lines) with time plotted on a logarithmic scale for ease of view. For this particular term in the subnetwork-expansion, as 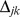 increases (and the dotted 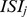 shifts to more closely resemble the solid 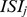) the var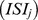 increases. The rate at which var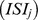 increases as 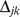 is increased is approximately 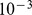 for this system (as indicated by the legend ‘var(ISI)+E-3’). A separate calculation can be performed which shows that the rate at which the sensitivity of the 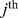 LNI (i.e., 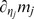) changes as 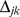 is increased is approximately 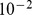 (as indicted by the legend ‘snstvty+E-2’). Thus, by strengthening the presynaptic-inhibitory connections from several other LNIs onto the 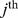 ORN-LNI pair (while simultaneously reducing 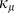 so as to maintain 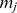), we can readily show that, to 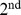-order, these shifts collectively increase both var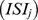 and the sensitivity 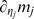. [B–G] In these panels we show similar plots illustrating the influence of various other subnetworks on the reliability of the 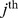 LNI. These plots use axes identical to those shown on the inset in panel-A. Listed in reading order, these subnetworks are denoted by 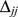, 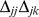, 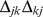, 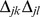, 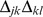, and 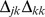. Note that the contribution of the autapse 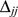 actually decreases var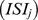, and the contributions of the 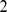-edge subnetworks all decrease the sensitivity 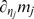 (albeit with magnitudes that are dwarfed by the contribution of the 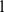-edge subnetwork 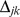).

Formally speaking, this analysis is nothing more than a Taylor-expansion of var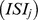 in terms of the coupling strengths of the network. Namely, var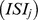 depends on many parameters (e.g., 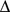, 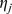, 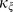, 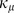), and if we assume that 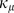 is implicitly dependent on 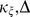 in such a way that 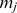 is constant, then we can Taylor-expand var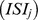 in terms of 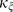 and the components of 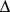. If we retain all terms up to second order, such an expansion has the form
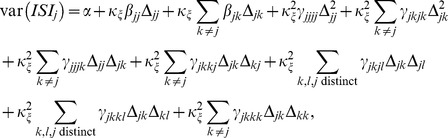
(3)


where 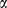 is a 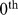-order contribution, each 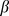 term corresponds to a 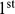-order contribution, and each 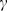 term corresponds to a 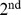-order contribution. Each 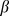 and 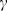 term is a correction to var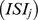 associated with a particular subnetwork containing the 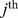 LNI (see Fig. 6 for an example). For example, the term 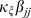 corresponds to the subnetwork in which the 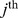 LNI (see Fig. 6 for an example). For example, the term 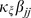 corresponds to the subnetwork in which the 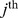 LNI presynaptically-inhibits its own ORN (the 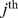 ORN) — 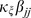 is the differential correction to var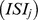 associated with increasing the connection strength 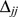, while appropriately decreasing 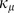. This correction 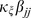 is actually negative (i.e., if one were to increase 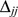, then the 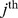 LNI would become more reliable). As another example, the term 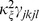 (with 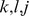 distinct) corresponds to the subnetwork in which both the 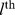 LNI and the 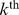 LNI presynaptically-inhibit the 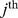 ORN. If both 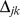 and 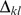 were to increase, then the change in var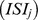 would be well-approximated by the change in the terms 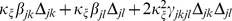 (in this manner, the 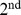-order correction 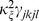 captures the change to var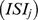 which is not accounted for by 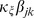 and 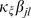). Indeed, given any specific network containing the 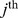 ORN-LNI pair, one can determine the effect of increasing 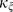 (or, equivalently, increasing all of the components of 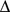 simultaneously) by dissecting the specific network and determining the contributions made by the various comprised subnetworks to var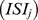. See Fig. 7 for an illustration of this technique.

**Figure 8:**
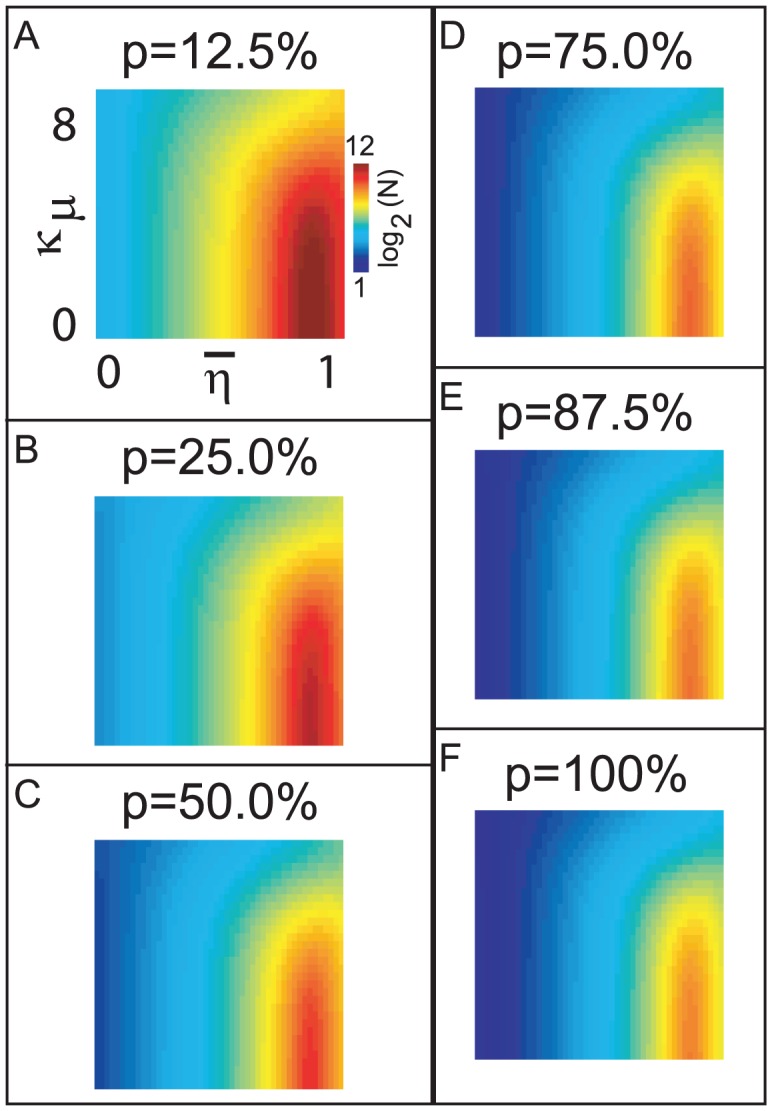
An analysis of sparsely-coupled Erdos-Renyi random networks, using a 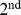-order subnetwork-expansion. Given a network of the form described in the section entitled “A population-dynamics approach towards verifying Hypothesis 2 within more general networks”, with 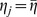 and 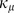 fixed, one may ask if, for the 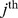 LNI, the reliability of this LNI would decrease if the presynaptic-inhibitory strength 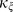 were to be increased (while simultaneously decreasing 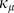 so as to maintain the firing-rate 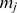). Let us denote this condition by ‘hypothesis-2.1’. By analyzing the terms in the 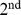-order subnetwork-expansion, one can readily conclude that hypothesis-2.1 holds if the 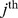 LNI does not presynaptically-inhibit its own ORN, and there is at least one other LNI which does presynaptically-inhibit the 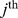 ORN. However, if the 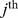 LNI presynaptically-inhibits its own ORN, then hypothesis-2.1 holds only if the size of the network is sufficiently large. This critical network size 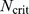 (above which hypothesis-2.1 holds with high probability) is a function of the background firing-rate of the ORNs 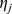, the strength of vesicle-depletion 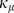, and the sparsity-coefficient 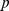 of the random network. In panel-A we plot 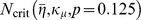, where we have calculated 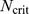 such that, for values of 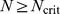, a randomly selected LNI within an E-R random network generated with sparsity-coefficient 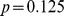 is highly likely (probability 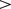
75%) to obey hypothesis-2.1, given that the LNI in question presynaptically-inhibits its own ORN. Values of 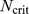 are displayed according to the colorscale shown on the right. In the remaining panels B–F we plot 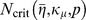 for different values of 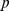. Note that, unless 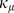 is small and 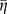 is large, it is highly likely that hypothesis-2.1 holds (even for LNIs which presynaptically-inhibit their own ORNs) for all LNIs within an E-R random network of size 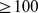.

By analyzing the various terms in this expansion, one can determine that by increasing the strength of certain elements of 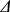, it is possible to actually lower var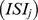, and make the 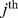 LNI more reliable. For example, by exclusively strengthening 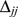 without increasing the other 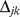, the reliability of the 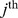 LNI would increase, in seeming contradiction to hypothesis-2.1. However, in a typical random network (containing many ORN-LNI pairs, and many presynaptic-inhibitory connections), the subnetworks which increase var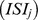 dominate those that lower var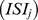, and thus a uniform increase in 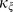 will increase var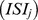. By analyzing the magnitudes of the various 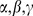 terms in Eq. 3, one can quantify this statement for any particular class of networks. For example, consider a random network of 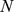 neurons for which each 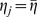, and each element of 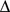 is independently chosen to be either 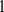 or 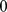 with probability 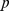 and 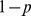 respectively (i.e., a Erdos-Renyi random graph with sparsity coefficient 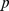). For any fixed LNI 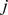, which does not presynaptically-inhibit its own ORN, there will be approximately 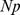 subnetworks of the form 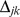, 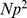 subnetworks of the form 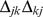, 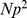 subnetworks of the form 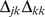, 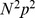 subnetworks of the form 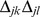, and 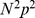 subnetworks of the form 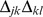 (where we assume 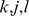 are distinct). If 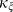 and 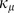 are modified for such a network so as to maintain 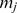, then as 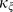 is increased var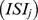 will increase as well (since the reduction in var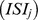 caused by the subnetworks of the form 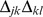 and 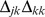 is more than cancelled out by the subnetworks of the form 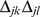). If, on the other hand, the 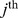 LNI presynaptically-inhibits its own ORN, then in addition to the various subnetworks mentioned in the previous case, there will be a single subnetwork of the form 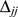, and approximately 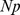 subnetworks of the form 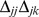. If 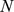,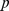 are not sufficiently large, then the contribution to var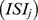 will be dominated by the 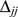 subnetwork, and increasing 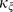 (while decreasing 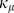 to maintain 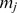) will actually reduce var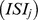. An example of the critical 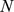 below which hypothesis-2.1 fails is shown in Fig. 8, which illustrates that hypothesis-2.1 holds with high likelihood for all LNIs within Erdos-Renyi random networks obeying the dynamics specified in Eq. 16 (assuming that each 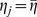), so long as 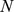 is sufficiently large.

This type of approach can also be used to analyze hypothesis-2.2. Using similar techniques as above, one can readily show that hypothesis-2.2 holds with high likelihood for all LNIs in an E-R random network of any size regardless of whether or not those LNIs presynaptically-inhibit their own ORNs, as long as their ORNs are presynaptically-inhibited by at least one other LNI.

Through the application of the above analysis, one can show that all sufficiently large E-R random networks obeying the dynamics of Eq. 16 have a high likelihood of satisfying the three properties associated with hypothesis-2 — namely, (0) there exists a 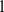-parameter family of network parameters ranging from type-A to type-B for which the firing rate of any particular LNI is maintained, (1) as the network parameters are shifted along this 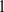-parameter family in the type-A direction, that LNI's reliability decreases, and (2) as the network parameters are shifted along this 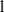-parameter family in the type-A direction, that LNI's sensitivity to its dedicated ORN channel increases. For the class of networks considered in Fig. 8 the critical 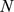 above which hypothesis-2 holds is roughly 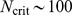. Although this critical value obtained from our idealized model should not be compared quantitatively with the number of LNIs in the real fly AL, we expect that a similar qualitative result should hold for more realistic models — namely that hypothesis-2 should hold for any model as long as the presynaptic-inhibitory network includes sufficiently many inter-glomerular connections and the number of LNIs is sufficiently large.

This type of subnetwork analysis can also be used to probe the relationship between connectivity and dynamics that exists in more complicated heterogeneous networks, including scale-free and small-world networks, as well as networks in which the different neurons are governed by different equations. In each case, one can use the distribution of subnetworks within the larger network to determine dynamical features associated with neurons inside that network. For example, one can easily show that, for a scale-free network obeying the dynamics specified in Eq. 16, if the 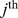 ORN-LNI pair has low incoming-degree and is presynaptically-inhibited by ORN-LNI pairs with high incoming degree, then the 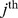 LNI is likely to violate hypothesis-2.1. This is simply because there will be an overwhelming number of subnetworks of the form 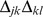, which each contribute to the decrease of var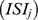 when 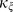 is scaled up (assuming as before that 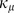 is appropriately decreased to maintain the firing-rate 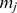).

Finally, before we conclude, we address the effect of excitatory interconnectivity in the AL, which was not taken into account in the simple model analyzed above. Let's assume for a moment that, in the above model, we had added excitatory cells (say, LNEs) which were also affected by the ORN input channels. When analyzing the effect of modifying 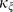 on the LNIs, the excitatory interactions associated with the LNEs only come into play at the 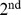-order. For example, if indices 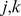 correspond to LNIs and index 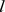 corresponds to an LNE, the simplest coupling terms by which LNE-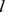 can affect LNI-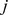 are of the form 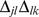. Similar to the 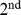-order contributions shown in Fig. 7, The contributions of this term are dwarfed by the contributions of the LNI-LNI terms 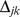 and 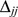. When analyzing the effects of modifying 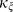 on the LNEs themselves, the situation is similar, with excitatory interactions playing a secondary role to the dominant contribution of first-order presynaptic-inhibitory connections. Thus, as long as the number of LNIs which affect each AL-neuron is not much less than the number of LNEs which affect that neuron (and as long as excitation and inhibition have effects of comparable magnitude), we expect the analytical results of this section to hold for networks which include both excitatory and inhibitory neurons.

## Discussion

Using our large-scale model for the fly antennal lobe, we have been able to put forth two hypotheses which concern the functional role played by synaptic-depression at the ORN synapses within the fly AL. The first of these hypotheses is that ORN
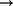
AL synaptic-depression may contribute to a form of variance coding when ORN firing-rates are high. The second hypothesis is that two network mechanisms which both give rise to synaptic-depression (namely presynaptic-inhibition and vesicle-depletion) participate in establishing a balance between the network's reliability and sensitivity.

The first hypothesis hinges on the fact that, as ORN firing-rates increase beyond the value at which PN firing-rates saturate, the corresponding increase in ORN
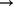
PN synaptic-depression diminishes the postsynaptic impact of each individual ORN firing-event. In many situations (e.g., when many ORNs stimulate each PN, and different ORNs fire independently of one another), this scenario directly implies that, as ORN firing-rates increase, the ratio between the variance in input to the PNs and the mean input to the PNs decreases. If the ORN
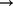
PN synaptic-depression is sufficiently large, the variance in input to the PNs can actually decrease as ORN firing-rates increase, while the mean input to the PNs remains the same. When the variance to the PNs decreases sufficiently, the PNs behave as though they are driven by an input current. This current-like drive typically gives rise to very regular (periodically firing) PN dynamics. This first hypothesis is very general, and evidence of this hypothesis manifests in our simulations for a wide range of parameter values. As mentioned above, synaptic-depression (including vesicle-depletion and presynaptic-inhibition) is just one of several mechanisms which could contribute to this variance-coding effect.

One of the most straightforward predictions of our first hypothesis is that, within the real AL, the trial-to-trial variability of PN responses should decrease as ORN activity increases beyond the point where PN firing-rates have saturated. This should be measurable at the level of individual PN responses, as long as the odor stimuli are chosen at the appropriate concentrations (see, e.g., [18]). Many of the experimental protocols which are currently employed are sufficient to test this prediction.

If such a variance code is indeed found in the fly AL, then the next question is whether or not the fly makes use of it. There are many ways in which an appropriately constructed downstream neuronal system could discriminate between two different stimuli that give rise to PN activity with similar firing-rates but differing degrees of regularity. For example, a downstream neuron may be wired to a subset of PNs in a given glomerulus in such a manner that (i) the steady nearly-periodic sequence of PN firing events associated with low-variance high-firing-rate PN activity (observed when 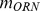 is very high) does not stimulate the downstream neuron sufficiently to induce firing, whereas (ii) the occasional clusters of PN firing-events which occur during high-variance high-firing-rate PN activity (observed when 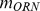 is not quite so high) do stimulate the downstream neuron to fire. This is one of many potential ‘readout’ mechanisms that could serve to discriminate amongst stimuli which generate high ORN firing-rates. Such a readout mechanism may be at work within Kenyon cells, which have been observed to act like coincidence detectors [26]–[28]. A Kenyon cell that has a ‘high-threshold’ for firing may function as a coincidence detector, while also responding preferentially to spike-clusters within high-variance high-rate PN activity, but not to low-variance high-rate PN activity. An illustration of this principle is given in Fig. 2H. There are many other potential readout mechanisms that could serve to detect changes in the variance of PN activity, and effective readout mechanisms are likely nonlinear in nature [29],[30].

Our first hypothesis may have significance for concentration-coding within the fly olfactory system. As an odor's concentration increases there are two typical changes to the ORN activity induced by that odor: (i) the number of activated ORN classes increases, and (ii) the ORN firing-rates within each activated class increase and eventually saturate [18], [31], [32]. It has been proposed that the firing-rates of the PN population across many glomeruli can collectively encode both stimulus identity and stimulus intensity [32]–[34], but it is still unclear how such a population firing-rate code could be deciphered by a downstream network within the fly. Our first hypothesis suggests an alternative to such a population firing-rate code, as the activity of a single glomerulus can also encode information regarding stimulus intensity, even when the glomerular firing-rates are saturated. Thus, a downstream decoder need not necessarily integrate information from multiple glomeruli in order to assess the intensity of a stimulus. In cases where a pair of high-concentration odors do in fact saturate the responses of most of the glomeruli which they target, typical readout mechanisms designed for rate-coding may not serve to discriminate these odors [35], and variance-coding mechanisms relying on transient bursts of activity (as mentioned above) may be more useful (see, for example, [36]).

The second hypothesis contains two statements. The first is that feedback-dominated synaptic-depression (i.e., presynaptic-inhibition) within type-A networks allows type-A networks to be more sensitive than type-B networks to shifts in input. Thus, type-A networks outperform type-B networks at odor-discrimination tasks when long observation-times are allowed. This statement seems to be true within our model network over a wide range of parameter values. The second statement is that feedforward-dominated synaptic-depression (i.e., vesicle-depletion) within type-B networks allows type-B networks to be more reliable than type-A networks (over multiple presentations of the same odor). This seems to be true within our large-scale model when (i) the typical number of ORN firing-events presynaptic to each PN within a glomerulus is larger than the typical number of LNI firing-events presynaptic to the ORNs which target that glomerulus, (ii) the vesicle-depletion experienced by the ORN synapses is relatively well stereotyped across ORN firing-events, and (iii) the presynaptic-inhibitory network includes many ‘inter-glomerular’ connections. These three conditions seem to hold for the fly AL [12], but in other situations these conditions may be violated, and the converse of this second statement may hold. For example, we conjecture that in a hypothetical system containing (i^′^) very many high-firing-rate LNIs and PNs driven by only a few low-firing-rate ORNs and (ii^′^) a vesicle-depletion mechanism that is highly variable from one ORN firing-event to the next, it is likely that LNI
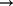
ORN presynaptic-inhibition can control the ORN input to the PNs much more reliably than vesicle-depletion of the ORN synapses could. In this hypothetical system type-B networks would be quite unreliable. Furthermore, even in a system with many ORNs and few LNIs, if (iii^′^) the presynaptic-inhibitory network is dominated by strong intra-glomerular connections (between LNIs and ORNs associated with the same odorant channel), then the subnetwork analysis carried out above indicates that type-A networks would actually be more reliable than type-B networks.

Obviously, the real AL for any given fly has a fixed architecture, and it does not seem likely that a fly could vary the effect of vesicle-depletion and presynaptic-inhibition on the ORN synapses to take advantage of a tradeoff between reliability and sensitivity. However, the hypothesis above does apply to the fly AL in the sense that fly physiology may be balanced to achieve some optimal compromise between gain (i.e., responsivity), reliability, sensitivity and discriminability over short observation-times.

### Suggestions for future study

The intuition gained in this study may be useful for understanding the coding properties of olfactory systems in other insects, or even in mammals, many of which also exhibit synaptic-depression.

In the olfactory system of many other insects, such as the locust and honeybee, the antennal lobe activity is characterized by oscillations which develop soon after the onset of stimulus. These oscillations are thought to be a key feature of the AL-response in these animals [26], [33]. Since such oscillations do not manifest quickly in the fly [17], the dynamical regimes studied in this paper are not of this nature. However, we can retune our computational model to produce oscillations by increasing the density of lateral connectivity within the AL (thus, bringing our model closer in structure to that of [33]). The analytical techniques used in this paper may also be useful for studying some of the phenomena associated with such an oscillatory regime.

Within certain mammals, such as the mouse, primary olfactory input to the olfactory bulb can be presynaptically inhibited by interneurons [37]–[39]). Because the architecture of the mammalian olfactory system is different from that of the fly, the hypotheses investigated in this paper may not directly apply. For example, in the mouse it has been found that inhibitory neurons in the olfactory bulb strongly presynaptically-inhibit the olfactory sensory neurons stimulating their own glomerulus, but not those stimulating other glomeruli [40], a situation markedly different from the presynaptic-inhibitory network of the fly AL [12]. Thus, as hinted in (iii^′^) above, one might expect type-A networks to be both more sensitive *and* more reliable than type-B networks in the mouse. The extra coding power afforded by ‘feedback-induced’ synaptic-depression in this scenario may be necessary for an animal which is forced to sample it's olfactory environment using short observation times (e.g., nose-pokes and sniffs). This sort of speculation begs for a more detailed investigation of the structural and dynamic mechanisms at work in the mammalian olfactory system.

## Materials and Methods

### Overview of large-scale point-neuron model

In this section we describe the point-neuron model we used to investigate the dynamics of the fly AL. This model incorporates 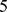 glomerular channels, each with 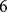 PNs, 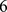 LNEs, 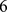 LNIs and 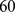 ORNs, and incorporates presynaptic-inhibition as well as vesicle-depletion (see Fig. 1). Each PN, LNE, LNI and ORN is modeled using single-compartment Hodgkin-Huxley type kinetics using standard sodium and potassium currents that give rise to fast sodium spikes [22] similar in shape to those observed experimentally [41].

#### Model synaptic currents

ORN, PN and LNE excitatory synapses and LNI GABAergic synapses are modeled by fast-activating synaptic currents [34], [42]. Excitatory and GABAergic transmission are both modeled via stereotyped instantaneous neurotransmitter release in response to a presynaptic action potential, with ORN synapses experiencing vesicle-depletion. Although there is evidence for a slowly activating inhibitory current in the honeybee [43], we are not aware of any similar evidence for such slowly activating inhibitory current in the fly AL. There is, however, evidence for both long-timescale GABA-B type inhibitory currents, as well as short-timescale GABA-A type inhibitory currents in the fly AL [11]. To account for these two timescales, both a long- and short-timescale inhibitory current are incorporated into this model — the relevant synaptic currents include fast excitation (nAch-type, timescale 5–10 ms), fast inhibition (gabaA-type, timescale 10–15 ms) and slow inhibition (gabaB-type, timescale 100–400 ms).

#### Model connectivity

Experimental observations indicate that all the ORNs that express the same odorant receptor gene have similar odor responses and project to the same glomerulus in the brain [18], [44]. Each PN receives direct ORN input from a single glomerulus [6], and thus all the ORNs and PNs corresponding to a given glomerulus constitute a discrete processing channel. We have designed the architecture of our model to reflect these experimental observations — our model ORNs and PNs only project to PNs and LNs associated with their own glomerulus.

Experimental observations also indicate that glomeruli are interconnected by a network of local interneurons. There have been several experimental results implying that the lateral connectivity between glomeruli in the AL has a strong inhibitory component, and that this inhibitory component is partly due to presynaptic-inhibition (i.e., LNIs synapsing on the ORN axons presynaptically) [12], [13], [41]. It has also been revealed that ORN-PN synapses are quite strong, and likely experience substantial synaptic-depression through vesicle-depletion [11]. Our computational model reflects these observations, and model LNEs and LNIs project to neurons both within and outside their own glomerulus. In addition, the model LNIs only affect the ORNs presynaptically. That is, deposition of neurotransmitter from LNIs onto ORNs only suppresses the efficacy of the synapse at the ORN axon (without suppressing the membrane potential at the ORN soma). In order to model vesicle-depletion, the efficacy of the synapse associated with each ORN is depleted each time that ORN fires [22].

There is debate as to whether or not there is a functionally relevant large-scale structure to the lateral connections within the AL, such as center-surround excitation/inhibition, or chemotopy [7], [45]. The lateral connectivity in our model network is structured so that the interconnections between ORNs, PNs, LNEs and LNIs are sparse and randomly determined. Thus, the results of our model may be expected to generalize to a variety of networks with a degree-distribution similar to that of an Erdos-Renyi random-graph. The connectivity of the network is encoded by a matrix 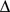, with 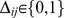 labelling the existence of a connection between cell 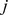 and cell 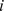. Each entry in the connectivity matrix 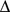 is randomly chosen to be either 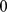 or 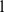 independently, with connection probabilities specific to the cell types and glomerular channel assignments of neurons 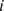 and 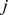. Both the connection probabilities, as well as the coupling strengths are chosen in a manner consistent with the literature (details given in the sections regarding benchmarking below).

#### Model odor input

The model ORNs themselves are each stimulated by Poisson input. In background (i.e., in the absence of an odor stimulus) the Poisson input rate is 350 Hz, and the input strength is low enough that the ORN background firing-rate is 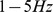, and the corresponding PN background firing-rate is 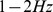 (consistent with experimentally observed firing-rates — [6], [41]). Odor presentation within this network is modeled by stimulating the ORNs corresponding to a subset of glomerular channels (typically around half of the glomerular channels) with additional high-rate Poisson input, in addition to the background Poisson input (consistent with experiments which indicate that an odorant typically activates multiple ORN types and triggers activity in 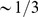 to 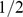 of the glomeruli — [18], [20], [46], [47]). In this manner, a model odor is represented in a combinatorial fashion — an odor is defined by the degree to which that odor drives the various ORN input channels. To simulate odors of different chemical composition, we stimulate different subsets of ORN input channels, whereas to simulate odors of the same chemical composition (but of differing concentration) we stimulate the same input channels to differing degrees, as motivated by the observation that varying the concentration of a given odor tends to modulate the firing-rates of responding ORNs in vivo [45], [48]–[50]. The odor-dependent noisy input signal is sufficient to drive individual ORNs strongly stimulated by the odor to firing-rates of 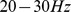. We choose a time-course of the odor-specific ORN current stimulus that is comparable to the time-course observed experimentally [18]. Specifically, at the time of ‘odor onset’ we increase the input-specific drive to the ORNs slowly (over 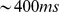), and at odor offset we decrease the input-specific ORN drive even more slowly (over 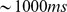). While there is some evidence of more complicated odor-specific temporal structure to ORN odor response, we will model only the temporally simplistic ORN response detailed above, so as to focus on emergent dynamics within the AL which manifest solely as a result of AL interconnectivity.

### Benchmarking the model

We have tuned the model so that, with a single set of parameters, the model exhibits a dynamic regime that is consistent with a variety of experimentally observed phenomena.

We attempted to ensure that the model network architecture is consistent with the literature. For example, motivated by [13], We chose the inhibitory postsynaptic coupling strengths from LNIs
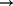
PNs so that the lateral inhibitory IPSC to a PN has both a fast and slow component (in our model IPSCs incorporate 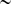
50% fast (gabaA type) and 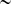
50% slow (gabaB type) inhibition). Similarly, we chose the inhibitory postsynaptic coupling strengths from LNIs
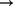
LNEs and LNIs
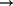
LNIs to be 100% fast-type. As another example, motivated by [41], we have chosen LNE
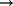
PN intra-glomerular coupling to be sparse enough (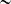
15%-25%) to align with the fact that direct LN
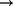
PN connections are rarely observed. Nevertheless, LNE
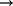
PN inter-glomerular coupling is dense enough that lateral excitatory input is still observed between most pairs of glomeruli [8], [9]. The lateral excitation between glomeruli is sufficiently strong that, even when ORNs belonging to a particular glomerulus are removed, some PNs and LNs within that glomerulus can still fire after odor presentation.

#### Stimulus driven dynamic transients reflect PN reliability and saturation

We tuned the ORN
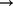
PN connection probabilities and connection strengths in our model so as to produce stimulus-triggered dynamic transients which are qualitatively similar to experiment. This stimulated set of ORNs directly activates a corresponding subset of glomeruli. As the glomeruli respond to the ORN input, the glomerular activity pattern shifts and spreads to include other glomeruli not directly stimulated by the odor.

**Figure 9:**
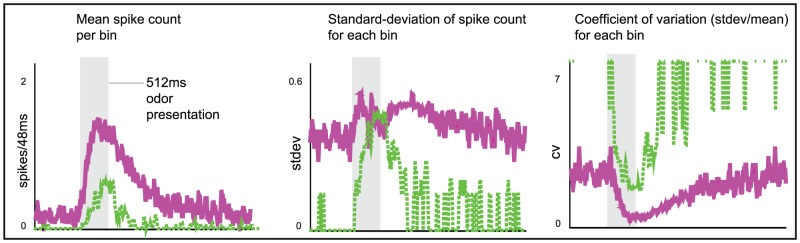
PNs are more reliable than their individual ORN inputs. Shown are averaged response curves for a typical model PN (magenta, solid) and model ORN (green, dashed) associated with the same glomerulus in our model. The grey overlay indicates the 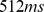 odor presentation period. Spikes were counted in 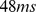 bins. The mean spike-count per 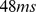 bin (averaged over 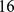 trials) is shown on the left. The standard-deviation in spike-count per 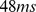 bin is shown in the center, and the coefficient of variation (standard deviation
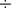
mean) is shown on the right. Note that, qualitatively similar to experiment [20], the model PN activates more quickly, has higher firing-rates, and is more reliable than the ORN.

**Figure 10:**
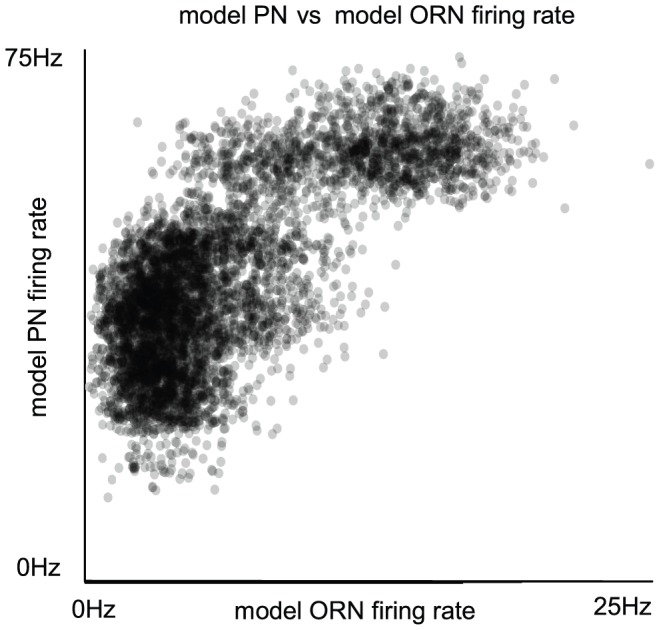
The relationship between ORN firing-rates and PN firing-rates is nonlinear. Shown is a scatterplot of model PN and model ORN firing-rates associated with a typical glomerulus in our model. Spike rates were measured during the 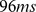 epoch during which PN firing-rates peak following odor presentation. Note that, qualitatively similar to experiment [20], the model PN firing-rates saturate for relatively small values of ORN firing-rates.

As shown in Fig. 9, the stimulus triggered firing-sequence of a typical model PN is more reliable than the corresponding sequence for a typical ORN associated with the same glomerulus, and the PN PSTH typically peaks after 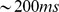 but before the ORN PSTH peaks (consistent with [20]). The sensitivity and reliability of PN activity within our model is critically dependent on three features: (i) the convergence ratio of ORNs to PNs must be sufficiently high that any given PN receives strong reliable input (summed over ORNs) immediately after odor onset, (ii) there must be some synaptic-depression at the ORN synapses, otherwise the PN activity does not peak before the ORN activity peaks (rather, the PN activity saturates and remains constant), and (iii) the combination of ORN activity and synaptic-depression at the ORN synapses must give rise to essentially ‘mean-driven’ (i.e., low-variance super-threshold) input to the PNs during odor presentation. These three network mechanisms seem to be necessary and sufficient to give rise to high-firing-rate PN activity which peaks prior to the peak in ORN activity, and which is more reliable than any single ORN's activity. We note that the source of synaptic-depression is not constrained by this particular phenomena — either vesicle-depletion or presynaptic-inhibition (generated by the recruitment of LNIs in other glomeruli) can give rise to mean-driven ORN
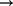
PN input, and hence to reliable PN firing sequences. As shown in Fig. 10, the PN firing-rate is a nonlinear function of ORN firing-rate when averaged across odors and trials and PN/ORN pairs within any given glomeruli, as is consistent with experiment [20].

#### PNs are more broadly responsive to odors than their ORN class would indicate

Experimentally, it has been observed that most PNs respond to multiple odors [45], [51], and are more broadly responsive than their input ORNs [9], [20] — there are many different PNs which respond to odors that do not stimulate their respective ORNs, and there are PNs which do not respond very strongly even when their respective ORNs are strongly stimulated. Thus, due to the lateral connectivity within the AL, the total set of activated glomeruli corresponding to a particular odor is generally not in one-to-one correspondence with the set of activated ORN olfactory receptors stimulated by that odor. Many believe that the inter-glomerular crosstalk is critically important for redistributing the glomerular activity within the AL ([41], but also see [7], [45]). It has been postulated that this re-expression of the odor at the glomerular level is advantageous for the fly, as the combinatorial code linking different odors to different glomerular subsets serves to separate similar odors more efficiently than the corresponding combinatorial code at the olfactory receptor level [20].

**Figure 11:**
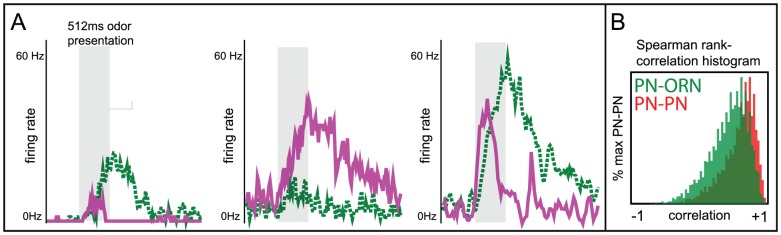
PNs exhibit broader odor responses than their associated ORNs. [A] Shown are trial-averaged firing-rate curves for various model PNs (magenta, solid) and associated model ORNs (green, dashed) in response to various model odors. Note that, qualitatively similar to experiment [12], the activity of the model PNs does not necessarily reflect the activity of the associated model ORNs. [B] Shown are the PN-ORN (green) and PN-PN (red) Spearman rank-correlation histograms for the model PNs and associated model ORNs (averaged over all PN and ORN pairs associated with each given glomerulus, and then further averaged over glomeruli — see [20] for the statistical methods used). Note that, qualitatively similar to experiment, the mean of the PN-ORN histogram is closer to 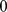 than the mean of the PN-PN histogram, indicating that, while PNs associated with a given glomerulus tend to respond to the same odors, they do not necessarily respond to the same set of odors which stimulate their associated ORNs.

We tuned the lateral connection probabilities and connection strengths within our model to produce a dynamic regime capable of rich glomerular activation patterns which are qualitatively similar to experiment. As shown in Fig. 11, the lateral connectivity in our model is sparse enough that oscillations do not develop during the initial odor-response, and yet strong enough that the activity of PNs and LNs within each glomerulus is not in direct correspondence with the activity of their respective ORN inputs. This lack of correlation between PN response and ORN response across odors can be quantified by measuring the PN-PN and PN-ORN rank-correlation, which is in good qualitative agreement with experiment [20]. In our model, as in experiment, LN activity is similar to PN activity (data not shown). For example, LN firing-rate can get quite high, and typically peaks shortly after odor onset [13].

#### ORN
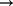
PN induced EPSCs attenuate as frequency of ORN synapse activation increases

**Figure 12:**
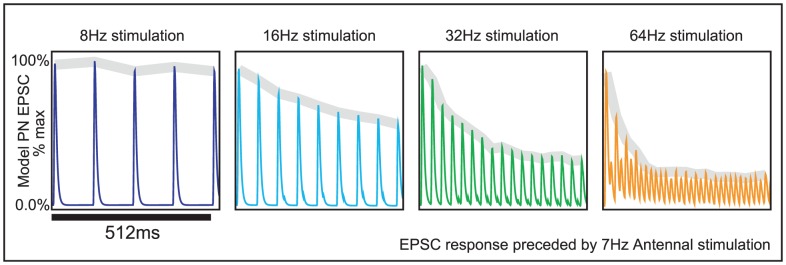
Synaptic-depression at the ORN synapses. Shown are current traces associated with a model PN in response to direct current stimulation of the model ORNs associated with that PN. Analogous to experiment [11], the model ORNs associated with the model PN have been stimulated by periodic 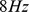 input current prior to the epoch shown in the figure. At the start of the epoch shown in this figure, the ORN stimulation is increased to 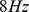, 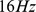, 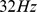, or 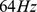. The trial-averaged model PN EPSCs in response these different stimulations are plotted (over a time interval of 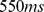). Above each EPSC curve, we show the envelope of the response in gray. This envelope is calculated by fitting a piecewise linear function to the maxima of the EPSC response sampled at the rate of stimulation. Note that, similar to experiment, the envelope of the PN EPSC attenuates more quickly when stimulated at 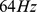 than when stimulated at 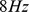.

Our model of vesicle-depletion and presynaptic-inhibition is phenomenological, and is intended to allow us to qualitatively reproduce and investigate the functional role of synaptic-depression at the ORN synapses. We have chosen a parsimonious model for vesicle-depletion, involving only one timescale of 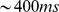, and one parameter 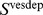. Similarly, our model of presynaptic-inhibition involves only the timescales of synaptic inhibition and a coupling strength 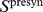 (see the section regarding vesicle-depletion and presynaptic-inhibition below). In order to ensure that our phenomenological model of synaptic-depression at the ORN synapses (a combination of vesicle-depletion and presynaptic-inhibition) was qualitatively accurate, we followed the experimental paradigm of [11]. We constructed a numerical experiment to measure the attenuation timescale of ORN
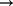
PN input (see Fig. 12). We first forced the ORN synapses to activate periodically at 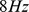 (to simulate ORN background firing-rates), and then once the system equilibrated to this periodic input, we increased the frequency of ORN stimulation (to 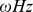) and measured the input current to each PN as a function of time. For a larger given frequency 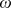, the attenuation of the PN EPSC will occur more quickly. We tuned the coupling strengths 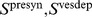 so that the attenuation time scale (as a function of 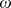) qualitatively matched experimental observations [11]. Our modeling work indicates that the attenuation time-scale match experiment as long as 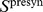 and 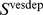 are sufficiently high (the exact ratio of 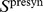 to 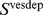 is not strongly constrained by this particular experiment).

**Figure 13:**
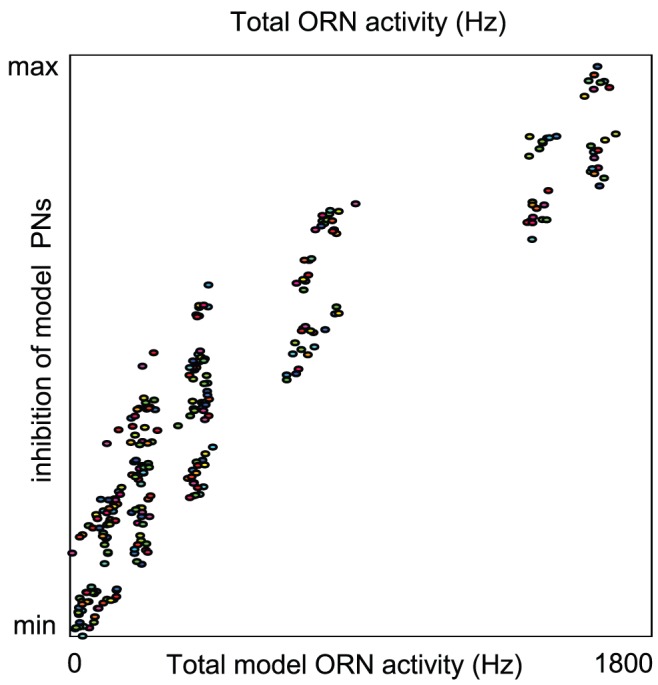
Presynaptic-inhibition is partly responsible for ORN 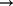
PN synaptic-depression. Shown is a scatterplot displaying the correlation between total ORN activity across all glomeruli in response to various odors, and the suppression of spontaneous EPSPs associated with a particular PN associated with a glomerulus which has been ‘shielded’ (i.e., the odor stimulus chosen does not affect the input drive to that glomerulus). In analogy with [12]. The PN suppression is measured as the difference in integrated PN membrane potential between (i) the scenario in which the PN receives spontaneous spikes from its associated ORNs in the absence of any odor, and (ii) the scenario in which the glomerulus associated with that PN is shielded and an odor is presented, in which case the activity generated within the other glomeruli reduce the effect of the spontaneous spikes impingent on the PN, and the spontaneous EPSPs are absent or greatly diminished. Note that, due to presynaptic-inhibition within the model, the correlation between PN EPSP magnitude and total ORN activity is qualitatively similar to experiment [12].

A further constraint on our model of synaptic-depression can be obtained by considering the correlation between total ORN activity, and reduction in subthreshold voltage observed at any given PN [12]. To obtain a roughly linear relationship between ORN activity and PN inhibition (as shown in Fig. 13), our model requires 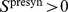.

### Details pertaining to Neuronal Model

The membrane potential of each ORN is governed by equations of the form
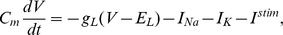
with stimulus current described in a section entitled “Odor Stimulation” below. The membrane potential for each PN, LNE and LNI is governed by equations of the form:
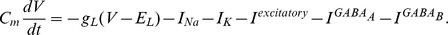
The parameters for the passive leak current are 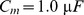, 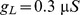, 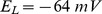.

#### Intrinsic currents

The intrinsic currents for each neuron consist of fast sodium and potassium currents 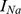 and 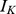. These currents obey equations of the following form:
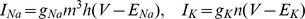
The maximal conductances are 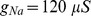, and 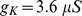. The reversal potentials are 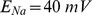, 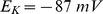.

The gating variables 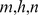 take values between 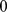 and 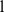 and obey equations of the following form:
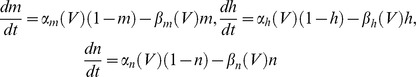


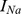
 and 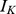 are described in [52]:
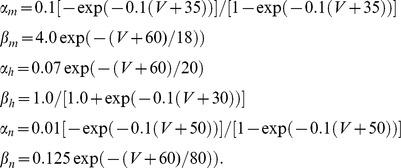



#### Synaptic currents

Given two connected neurons in the network, the synaptic conductances of the postsynaptic neuron are increased whenever the presynaptic neuron's membrane potential rises above a threshold of 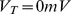 (i.e., when the intrinsic currents of the presynaptic neuron generate an action potential). The excitatory-synaptic current associated with the 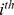 neuron in the network is governed by an equation of the following form:
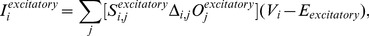
(4)

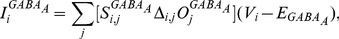
(5)with the excitatory reversal potential 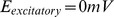. The efficacy of the excitatory-type synapse 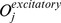 associated with the 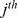 neuron obeys the equation:
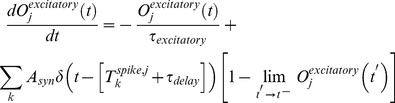
(6)where 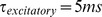, the times 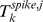 are the spiketimes of the 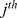 neuron, 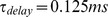, and 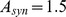 (adapted from [33], [34], [53], [54]). The term 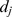 represents synaptic-depression at the ORN synapses, and is used to model both vesicle-depletion and presynaptic-inhibition at these synapses. This term 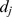 is identically 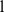 if 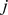 corresponds to a PN, LNE or LNI. The dynamics of 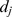 when 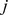 corresponds to an ORN will be discussed later. The differential equation Eq. 6 is structured so that 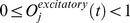 for all time, and in the absence of firing 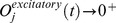. The synaptic GABA-A current for the PNs, LNEs and LNIs obeys equations analogous to Eqs. 4,6, with 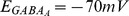. The efficacy of the GABA-A-type synapse 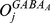 associated with the 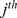 neuron obey the equation
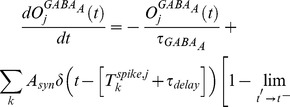
(7)with 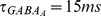. The synaptic GABA-B current for the PNs, LNEs and LNIs obeys an equation of the following form:
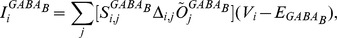
with inhibitory reversal potential 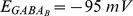. The quantity 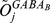 for the 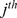 neuron obeys the equation:
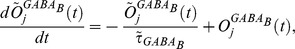
with 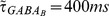. The efficacy of the synapse 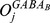 for the 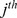 neuron obeys an equation analogous to Eq. 7, with 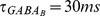. Thus, the slow GABA-B type synaptic current has a rise and decay time-scale, whereas the fast excitatory and GABA-A type synaptic currents only have decay time-scales (again adapted from [33], [34], [53], [54]).

The synaptic coupling strengths 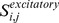, 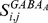, and 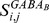 depend only on the cell types of neurons 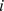 and 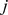, and are chosen so that only presynaptic ORNs, PNs and LNEs give rise to excitatory-type currents, and only LNIs give rise to GABA-A and GABA-B-type currents. The strengths are given by the following arrays:
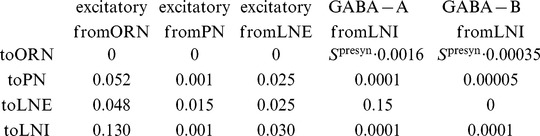
(8)


The parameter 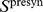 varies between 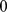 (no presynaptic-inhibition) and 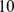 (strong presynaptic-inhibition). Note that the PN
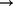
PN and LNI
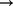
PN coupling strengths are all negligible, to account for the experimental observations that PNs may not be targeted by other PNs, or by local inhibitory interneurons. However, we do allow for PNs to connect to LNEs (and LNIs), as observed in [41]. If these strengths are set to 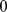 we can retune the remaining connectivity strengths so that our major conclusions still hold (data not shown).

#### Network connectivity

The intra-glomerular connection probabilities are given by the array:
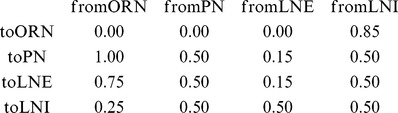
(9)and the inter-glomerular connection probabilities are given by the array:
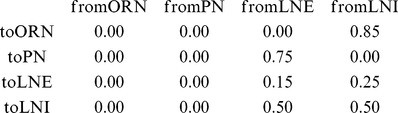
(10)


#### Modeling vesicle-depletion and presynaptic-inhibition at the ORN
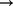
PN and ORN
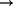
LN synapses

The ORNs in this model do not directly experience synaptic conductances from either the PNs or the LNEs. The excitatory-type from PNs and LNEs onto ORNs is identically 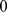 (see the array shown in (8) above). The GABA-A and GABA-B-type conductances associated with an ORN (say, with index 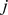) alter the term 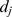 in Eq. 6, thus affecting the efficacy of synapses from the 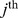 ORN onto the AL. The evolution of 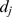 in our model is governed by two parameters: 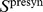 (described above), and 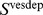 (described below). These two parameters will allow us to consider a 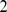-parameter family of model networks in which the strength of vesicle-depletion and presynaptic-inhibition can be altered independently (see Fig. 4). The equations governing the evolution of 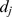 are
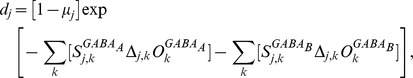
with the vesicle-depletion parameter 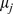 obeying the differential equation:
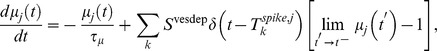
with 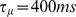. The parameter 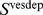 varies from 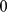 (no vesicle-depletion, 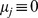) to 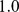 (complete vesicle-depletion with each firing-event). As the vesicle release rate per synaptic event is likely quite high within the real fly AL [11], values of 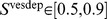 are most reasonable from a physiological standpoint. Note that the vesicle-depletion parameter 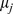 is bounded between 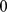 and 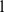.

#### Odor simulation

The stimulus current to the 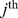 ORN is governed by the equation
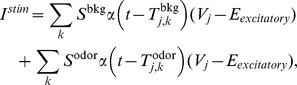
where 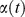 is a response-function such that 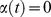 if 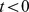, and 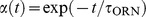 if 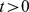, with 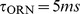. The spiketimes 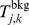 are drawn from a Poisson-process with rate 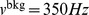. The strength of this background input is 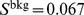. The spiketimes 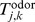 are drawn from a Poisson-process with rate 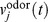 which depends on the time since odor onset, the odor being presented, as well as the ORN under consideration. Typical values for 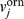 range from 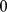 (when the odor does not directly stimulate the 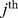 ORN) to 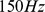 (when the 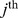 ORN is being strongly stimulated by the odor). The strength of this odor-specific input is 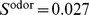.

The time-dependence of the odor-specific input-rate 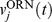 is governed by the factor
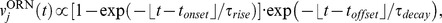
with 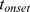 and 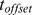 representing the onset and offset times of the odor stimulus (respectively), and with 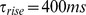, 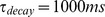. A typical odor (stimulation of the ORNs within 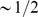 of the glomerular channels) activates 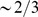 of the PNs, which fire at about 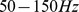. Not all of the PNs exhibit increased activity upon odor stimulation — a given odor will typically cause a few PNs which are not directly stimulated to actually decrease in activity (as a result of inter-glomerular inhibition). On presentation of a typical odor, the typical PN PSTH rises very quickly, and peaks after 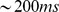, before the ORN PSTH peaks (at 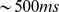 by construction). The typical PN PSTH decays more quickly than the ORN PSTH (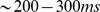 vs 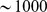 ms). Due to lateral connectivity, PNs respond to a broader selection of odors than their ORN inputs and most PNs have a ‘rank order’ of odors that is different from the rank order of their ORN inputs. Note that PNs exhibit a rise in firing-rate after odor offset only very rarely. This is a consequence of the fact that the current model only incorporates odors which increase the firing-rate of the stimulated ORNs. It has been observed that some odors actually decrease the firing-rate of certain ORN types, with perhaps a resurgence of ORN firing-rates after odor offset (as shown in DL1 response to cis-3-hexen-1-ol, cyclohexane and ethyl acetate — [20]). These types of inhibitory Odor
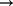
ORN responses could potentially give rise to richer PN dynamics (potentially triggered by PNs/LNs which fire greatly after odor offset), and these phenomena will be studied in more detail in future work.

#### Odor discrimination

In order to estimate the model network's ability to discriminate different odors, we measure the odor-dependent probability distribution of each PN's firing-rate, and use standard methods from classification theory [55]. For completeness we describe our procedure applied to firing-rate vectors.

Assume that we are estimating the network's ability to discriminate between 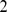 different odors. For each 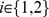, we perform multiple trials of odor 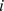, and estimate the probability distribution
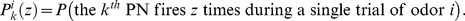
Once the 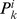 are sufficiently well estimated, we perform and classify individual odor trials. A single trial of odor 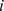 (randomly chosen to be either 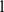 or 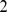 with 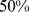 probability) will give rise to a vector 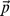 such that 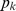 is the number of firing-events produced by the 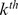 PN during that trial. By looking at a fixed 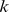 and comparing 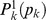 and 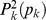, we can use the 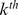 PN to identify a possible candidate stimulus (either odor 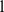 if 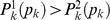, or odor 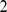 if 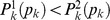). This process can be performed for each 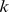, and in this way each PN ‘votes’ for a candidate stimulus. We tally these votes, weighting each one by the log of the information ratio associated with each PN. We use the weighting
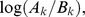
where 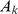 is the ‘hit-rate’ associated with the 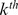 PN:
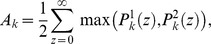
and 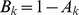 is the ‘error-rate’. This particular weighting is chosen so that 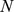 votes for stimulus 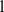 with error-rate 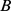 have the same combined weight as a single vote for stimulus 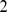 with a far smaller error-rate of 
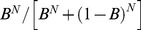
. The sum of the weighted votes is compared to determine the candidate stimulus underlying this particular trial. If the candidate stimulus matches the true stimulus, the trial is classified correctly. If not, the trial has been classified incorrectly. By going through this process with multiple trials, we can generate a probability that any given trial will be classified correctly. To perform 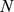-way discriminability tasks, we go through an analogous procedure, performing all 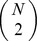 pairwise discriminability tasks for each sample observation, and ultimately selecting the candidate stimulus corresponding to the majority (with ties automatically counted as incorrect).

We have chosen this particular procedure because it allows us to take advantage of components of the firing-rate vector which carry substantial information (as measured by the information ratio), without requiring an estimate of the joint distribution of firing-rates (across PNs) for any particular odor. Thus, this measure of discriminability is more sensitive than typical linear discriminators which use the Euclidian distance between firing-rate vectors (see [20]), but does not succumb to the curse of dimensionality associated with the large number of distinct PNs.

### An idealized model used to illustrate variance coding

Here we describe in detail the idealized model used in the section entitled “A simple cartoon of variance coding” in the main text. This model includes a single conductance-based integrate-and-fire PN, driven by a set of 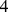 ORNs, each endowed with a simple model of synaptic-depression. Each of the 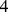 ORNs is modeled as a Poisson process with fixed rate 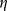 (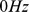 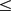 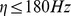). The coupling strength 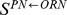 between the ORNs and the PN is modulated by a term 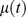 (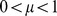), which is intended to model vesicle-depletion at the ORN synapses. As each ORN fires, this 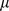 term will give rise to synaptic-depression between the ORNs and the PN.

The membrane potential 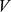 and conductance 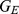 for this single PN obey the following differential equations:
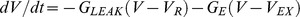


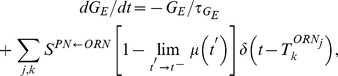
where 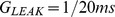, 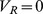 is the reset potential, 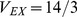 is the excitatory reversal potential, and 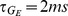 is the conductance time-constant. The voltage 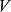 evolves continuously until 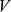 reaches a threshold 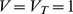, at which point the PN fires, and 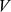 is reset to 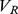. The conductance 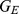 evolves continuously except when an ORN (say, the 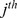 ORN) spikes, at which point 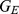 jumps. The time 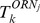 is the 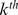 spiketime of the 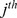 ORN, and 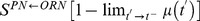 is the coupling strength associated with the 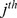 ORN at time 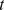. If 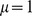, the synapses between the 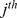 ORN and the PN are 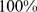 exhausted. If 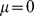, the synapses between the 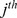 ORN and the PN are completely refreshed. For this simple model 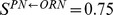, and the equation for 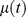 is given by:
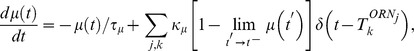
where 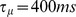 is the time-constant associated with vesicle-depletion. The term 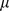 decays to 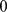 continuously, except when the 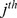 ORN fires at some time 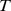, at which point 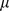 jumps by an amount proportional to 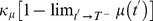 (the limit 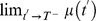 is used since 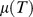 is not technically defined). The parameter 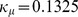 governs the relative increase in 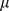 associated with each spike 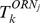, and hence 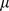 is bounded between 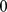 and 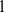. The parameters 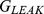, 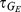 and 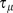 are chosen to be consistent with typical point-neuronal models, and the parameter 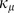 is chosen essentially arbitrarily (different choices for 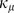 do not qualitatively change the results).

### A simple analyzable cartoon of variance coding

As a simple cartoon which illustrates Hypothesis 1, consider a single PN modeled by a conductance-based integrate-and-fire neuron [22], driven by a single ORN modeled as a Poisson process (with firing rate 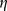). The state variables of the PN are the membrane-potential 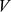, the excitatory conductance 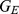, and the vesicle-depletion parameter 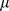. The equations governing the state of the PN are
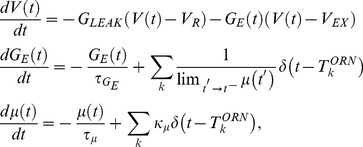
(11)where 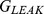 is the leakage conductance, 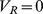 is the reset potential, 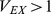 is the excitatory reversal potential, and 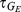 is the conductance time-constant. The voltage 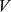 evolves continuously until 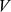 reaches a threshold 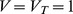, at which point the PN fires, and 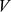 is reset to 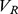. The conductance 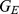 decays to 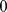 continuously except when the ORN fires. The time 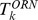 is the 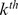 spiketime of the ORN, and is produced by a Poisson process with rate 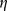., The conductance 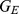 jumps by 
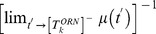
 at time 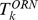 (the limit is used since 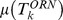 is not technically defined). The vesicle-depletion parameter 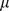 decays to 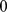 continuously with time-constant 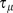, except when the ORN fires, at which point 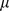 jumps by 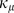. In this simple model the vesicle-depletion parameter 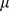 and conductance 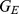 are both bounded to lie within 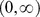. As the vesicle-depletion parameter 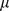 increases, the effect of ORN spikes on the PN conductance decreases. It should be noted that the functional form for the ORN
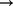
PN synapse — in this case modeled by 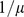 — is chosen to make the subsequent analysis easier, and is not particularly realistic (as very small values for 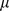 imply a very strong ORN
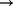
PN synapse). Nevertheless, the general picture implied by this cartoon holds for more realistic models of vesicle-depletion (see the section entitled “A simple cartoon of variance coding” in the main text).

The simple model Eq. 11 can be analyzed by considering the long-time evolution of the PN. For sufficiently small 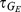, 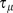, 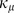 and sufficiently large 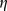 (with 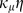 fixed), it can be shown [56] that the equilibrium-distribution of 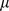 (collected by sampling over a sufficiently long time interval) is well-approximated by a Gaussian, with mean 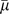 and standard-deviation 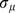 given by:
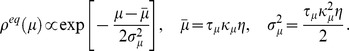
It can also be shown that, under these conditions, the equilibrium-distribution of 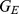 is also well-approximated by a Gaussian, with mean 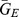 and standard-deviation 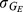 given by:
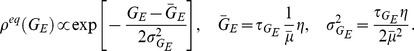
Using the expression for 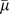, the expressions for 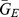 and 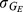 can be simplified to
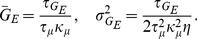
Thus, for a sufficiently small 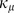, as 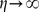 the variance 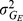 of equilibrium-distribution 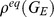 shrinks to 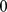, and the mean 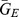 remains constant. Thus, as 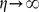, the long-time conductance-distribution becomes sharply peaked around 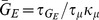; so much so that, for sufficiently large 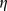, the PN effectively has a fixed excitatory-conductance 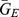 and will fire perfectly regularly with a period of
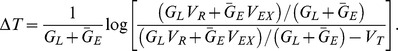
The excitatory conductance 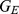 is, in this case, independent of the activity of the PN because the ORN input is only affected by vesicle-depletion, and not by presynaptic-inhibition. Nevertheless, the conclusions we draw from this simple model are quite general, and will hold for more realistic models of synaptic-depression.

Note also that synaptic-depression is critical to hypothesis-1 within this model. If 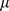 were fixed to be 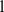 (i.e., no synaptic-depression of the ORN synapses), and 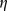 were sufficiently large, then the equilibrium-distribution of 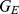 would be Gaussian with a mean and variance that grow unbounded as 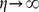.

### A simple model illustrating the tradeoff between reliability and sensitivity

In this section we analyze the model used in the section entitled “A simple analyzable cartoon of the tradeoff between reliability and sensitivity”.

To analyze the solutions of Eq. 1, let's assume for the moment that 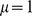, and 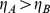 (i.e., neuron 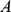 fires more frequently than neuron 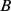). If 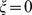, then 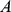 and 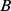 do not affect one another. The steady-state firing-rate 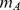 of neuron 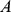 is 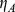, and the steady-state firing-rate 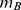 of neuron 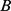 is 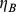. Let us define 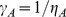 and 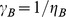. Since both 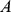 and 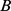 are perfect phase-oscillators, 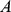 and 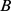 fire perfectly regularly every 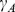 and 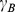 time-units (respectively). If there is a difference in ORN inputs to these two neurons (say, 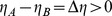), then the difference in firing-rates is 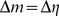. Thus, if 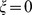, this system is perfectly reliable (in the sense that the ISI distribution of 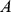 and the ISI distribution of 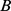 both have 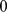 variance), and somewhat sensitive to shifts in the input (in the sense that any difference 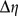 in input is reflected in the difference 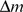 of the output firing-rates).

If 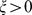, then 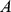 and 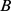 affect one another with several consequences: (i) the steady-state firing-rates 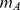 and 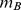 will be lower than 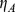 and 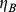 (respectively), (ii) the ISI distributions of 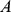 and 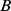 will have nonzero variance, and (iii) the difference in steady-state firing-rates 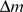 will be greater than 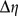. Indeed, as 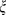 increases away from 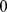 the system becomes less reliable while becoming more sensitive to shifts in the input. More specifically, for a given fixed 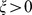, the system will settle down to a steady-state dynamics in which neuron 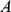 fires either 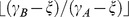 or 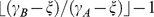 times in between each pair of 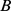-firing-events. The steady-state sequence of spike-times is independent of the initial state of the network and, while not generally periodic, can be solved for explicitly.

**Figure 14:**
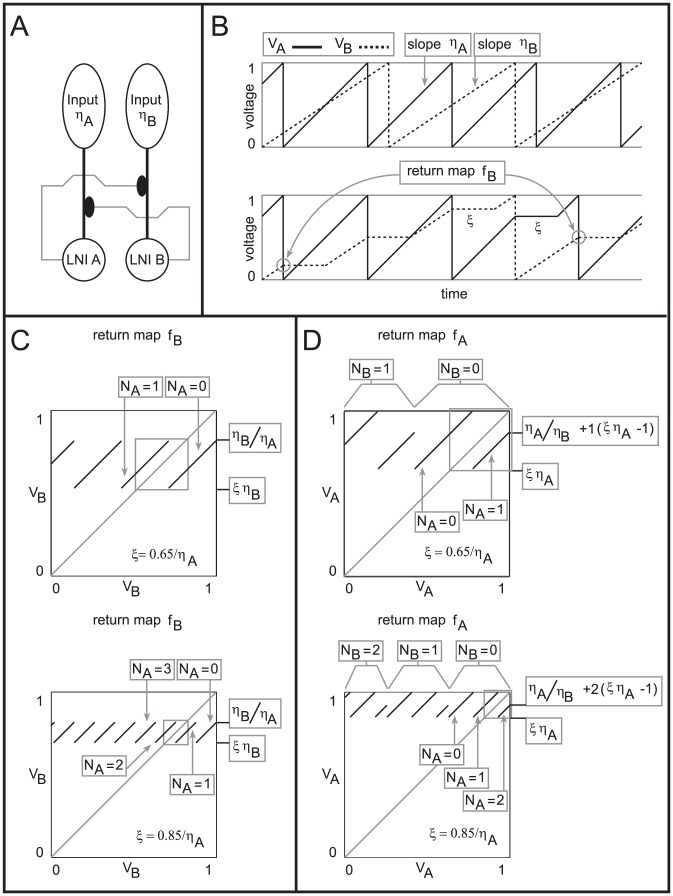
A simple analyzable cartoon of the tradeoff between reliability and sensitivity. [A] Shown is a schematic of the simple network, consisting of 2 ORN
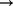
LNI pairs, each of which presynaptically inhibits the other. [B] Shown on top are sample voltage-traces for the two LNIs (represented by 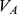 and 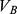) for the case 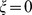. Shown on the bottom are sample voltage-traces for the two LNIs in the case that 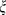 is nonzero. Note that after LNI A fires, 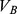 is constant for 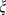-time. Similarly, after LNI B fires 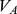 is constant for 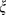-time. A pair of voltages for LNI B are circled. This pair of voltages 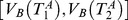 corresponds to a point on the graph of the return map 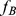, namely 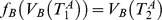. For this point on the graph of 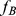, 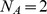, and 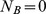. [C] Shown on the top and bottom are return maps 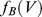 for the values 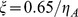, and 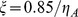, respectively. [D] Shown on the top and bottom are return maps 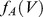 for the values 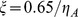, and 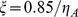, respectively.

To show why this is true, we consider the return-maps 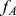 and 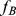. We define the return-map 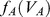 as follows: given a spike of neuron 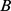 (say, 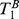), let 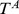 be the first spike of neuron 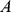 which occurs after 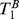, and let 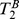 be the first spike of neuron 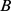 which occurs after 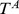 — we define 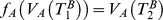. Similarly, given a spike 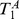 of neuron 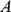, let 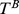 be the first spike of neuron 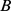 after 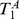, and let 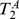 be the first spike of neuron 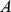 after 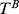 — we define 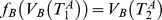. For the return map 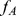, we can also define the numbers 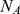 and 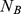 as follows: 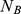 is the number of times 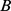 fires in between 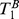 and 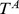, and 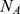 is the number of times 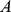 fires in between 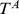 and 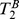. Similarly, for the return map 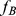, we can define 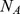 as the number of times 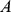 fires in between 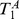 and 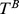, and 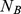 as the number of times 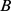 fires in between 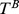 and 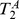. See Fig. 14 for an example of these return maps.

Recall that, without loss of generality, we have assumed 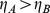. By considering the return maps 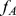 and 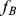, one can easily show that the maximum and minimum of 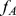 are 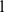 and 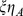, respectively, and that the maximum and minimum of 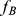 are 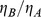 and 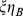, respectively. Moreover, 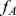 maps the interval 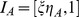 into 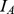. Similarly, 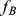 maps the interval 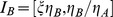 into 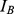. It is straightforward to show that the image of 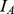 under 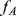 is composed of 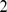 sub-intervals: (i) an interval of length 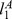 for which 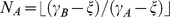, and (ii) an interval of length 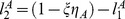 for which 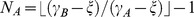. Similarly, the image of 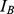 under 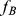 is composed of 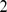 sub-intervals: (i) an interval of length 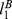 for which 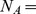 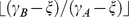, and (ii) an interval of length 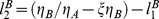 for which 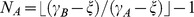. Letting
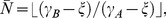
(12)one can show that the lengths 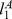 and 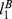 are given by
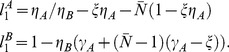
(13)For both the sub-maps 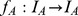 and 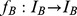 the number of extra spikes 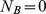. These observations allow us to conclude that the steady-state ISI distribution for neuron 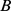 (i.e., 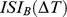) has a peak of magnitude 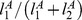 at 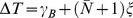, and a peak of magnitude 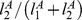 at 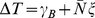. Similarly, the steady-state ISI distribution for neuron 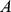 (i.e., 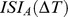) has a peak of magnitude 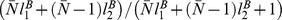 at 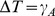, and a peak of 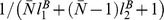 at 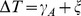. The steady-state firing-rates associated with these ISI-distributions can be expressed in closed form and directly computed:
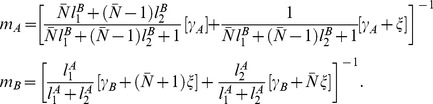
(14)Similarly, the means and variances associated with these steady-state ISI-distributions can be expressed in closed form (see Fig. 5).

By considering these expressions for small 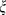, one can see that the steady-state return-map 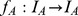 consists of segments of length 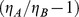 and 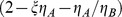, corresponding to inter-spike-intervals for neuron 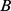 of length 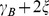 and 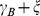, respectively. Thus, 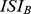 has 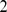 distinct peaks (at 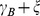 and 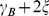, respectively), and as 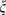 increases the distance between these two peaks increases. As a consequence, as 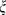 increases, the variance in 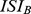 increases. In effect, a larger 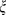 implies that extra spikes from 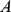 have a larger effect on the 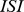 of 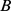. A similar argument applies to 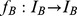 and 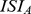, and one can also show that, as 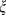 increase the difference in firing rates 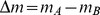 also increases (see Fig. 5). Thus, within this simple network, presynaptic-inhibition between the neurons disrupts their natural regularly-firing behavior, and increases the variance of their ISI distribution (thus decreasing their reliability).

In the discussion above, we assumed that 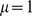. If we assume 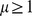, we can express the system firing-rate 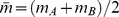 in closed form as a function of 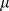,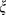 (simply by replacing 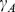 with 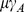 and 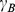 with 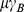 in Eqs. 12,13,14). By requiring the system firing-rate to be constant, we can define 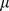 implicitly (as a function of 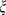,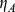,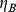 and the system firing-rate 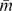). Thus, we can directly compute the 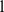-parameter family of networks which, for fixed 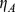,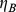, attain a fixed system firing-rate 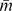. As shown in Fig. 5, this 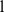-parameter family of networks does indeed range from type-A networks (with high 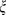 and low 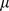) to type-B networks (with low 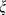 and high 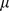). Moreover, as one moves along this 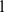-parameter family of networks by increasing 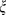 (and decreasing 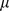 appropriately), the variance in 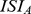 and 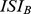 increases, and the sensitivity 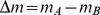 also increases. In conclusion, this simple network illustrates that presynaptic-inhibition is capable of increasing the variance of the ISI distributions of the neurons within that network (hence reducing their reliability), while at the same time increasing the sensitivity of the neurons' firing-rates to subtle shifts in input.

### Analysis of signal-to-noise ratio in a general discrimination task

In this section we provide details regarding the analysis in the section entitled “A simple cartoon of optimizing discriminability over short observation-times”. Our goal is to determine from a measurement 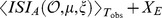 whether the input to the system is 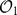 or 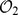. Let us denote by 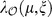 and 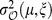 the mean and variance of 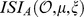. As long as 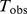 is sufficiently large, the estimate 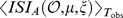 can be considered to be drawn from 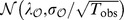. Thus, as long as 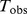 is sufficiently large, the measurement 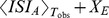 can be considered to be drawn from 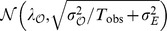 (since 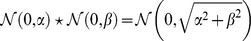). If we attempt to discriminate between the two possible inputs by using a linear-classifier, then the error 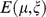 associated with the best linear-classifier is simply given by the overlap of the distributions 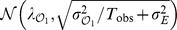 and 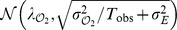. Because 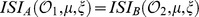 and 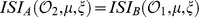 for this simple scenario, and the variance of these 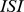 distributions is very similar for 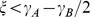 (see Fig. 5), the error 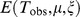 is well-approximated by
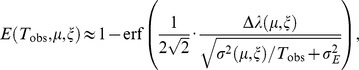
where 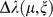 is the difference in the means of 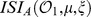 and 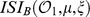, and 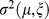 is the average variance of 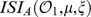 and 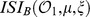.

### A discrete state model used to analyze hypothesis-2 within general networks with arbitrary architecture

In this section we describe the point-neuron model used in the section entitled “A population-dynamics approach towards verifying Hypothesis 2 within more general networks”. This model is a stripped down version of the fly AL, consisting of 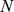 discrete-state LNIs, each driven by a different ORN. We will model each ORN-LNI pair as a discrete-state discrete-time Markov process which is as simple as possible, while still retaining the following features: (i) each LNI generates spikes, (ii) each ORN input spike contributes to the vesicle-depletion of that ORN
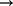
LNI synapse, and (iii), each LNI spike gives rise to presynaptic-inhibition of ORN
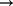
LNI synapses. We will model the 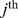 ORN-LNI pair using the state-variables 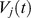, 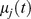 and 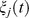 which represent LNI membrane-potential, ORN vesicle-depletion and ORN presynaptic-inhibition, respectively.

At each discrete time, each state variable is either 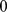 or 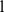, thus, at each time, the 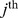 ORN-LNI pair is in one of 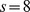 states. The input from the 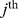 ORN to the 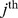 LNI is modeled as a bernoulli-random-variable 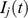, which is 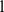 with probability 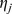 (and 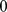 otherwise). The state-variables undergo transitions of the following form: 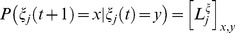, 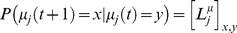, 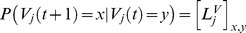, where
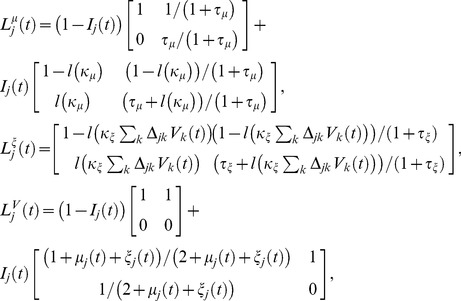
(15)and the function 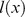 is the logistic function
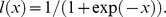
For this system 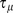 and 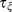 are the typical persistence times of the 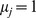 and 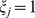 states (respectively), and we will assume that 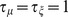. The 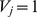 state is considered a ‘firing’ state for LNI 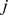. If 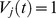, then 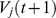 always equals 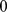. If 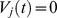 and the 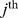 LNI does not receive input (i.e., 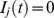), then 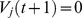. However, if 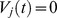 and 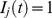, then 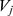 may transition to the firing state. Given that 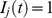, the probability of 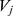 transitioning from 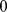 to the firing-state is typically 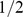, but is lowered if either 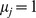 or 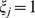. The vesicle-depletion parameter 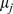 is likely to transition to the 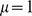 state whenever the 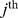 LNI receives input (i.e., 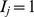). The presynaptic-inhibition parameter 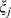 is likely to transition to the 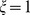 state whenever many other LNIs in the network fire. Note that the connectivity matrix 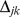 encodes the connectivity of the network, and can be chosen to encode many different network architectures (e.g., a densely connected homogeneous network, or a sparsely connected heterogeneous network). If 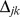 is nonzero, then the 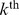 LNI presynaptically-inhibits the 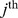 ORN, making it more likely that 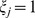, and thus less likely that ORN input from the 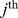 ORN to the 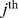 LNI will cause the 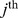 LNI to fire. For this model 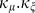 are the overall strengths of vesicle-depletion and presynaptic-inhibition. As 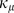 increases, the likelihood of 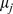 transitioning to the 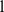 state increases. Similarly, as 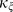 increases, the likelihood of 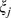 transitioning to the 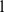 state increases as long as 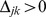 for some 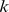. Ultimately, we will assume that the probability that the 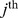 neuron will transition from the state 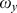 = {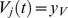, 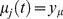, 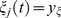} at time 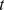 to the state 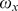 = {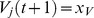, 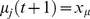, 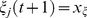} at time 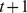 is given by
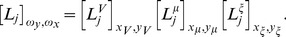
(16)Note that 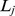 is an 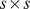 state-transition matrix which depends on the state of the 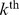 neuron in the system as long as 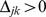.

### Analysis of reliability and sensitivity using a subnetwork expansion

This section reviews a diagrammatic approach to analyzing network dynamics, and presents the salient calculations relevant to analyzing ISI-distribution and firing-rate (which can then be used to analyze reliability and sensitivity, respectively). One way to understand the equilibrium dynamics of a network such as Eq. 15 is to first picture the network as a point in phase-space 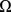, with the network's current state determined by the collection of parameters
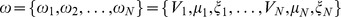
at the current time, where we denote by 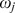 the state of the 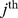 ORN-LNI pair. As time passes this network will trace out a trajectory 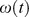 in phase-space, and this trajectory will depend on the network's architecture (i.e., 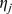, 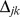, 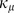, 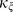). If one could determine the ‘typical’ phase-trajectories exhibited by this network (over very long times) then, in particular, one could determine this network's spike-time reliability. If one could determine how this network's typical trajectories shift as the input 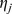 changes, then one could determine this network's sensitivity. The typical trajectories of a network can be determined by considering both the evolution-operator of the network 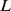, and the frequency 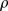 with which the network visits each part of phase-space (i.e., the network's equilibrium-distribution). The full evolution-operator 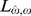 is the probability that the network moves from state 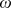 to state 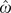 over one timestep. In this case 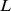 is an 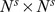 matrix such that each entry has the form
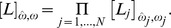
The probability that the network will be in state 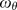 at time 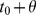, given that the network was in state 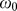 at time 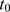 is
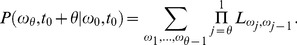
(17)
Eq. 17 can be thought of as an integral over all possible paths in state-space connecting 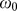 to 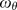 (i.e., each path traverses the system-states 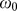, 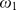, …, 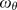 in sequence). The equilibrium-distribution 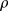 is an eigenfunction of 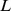 with eigenvalue 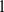 — namely
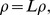
and in this case 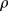 is an 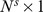 matrix (i.e., an 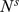-dimensional vector). In this discussion we will assume that 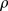 is unique (i.e,. 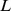 only has a single equilibrium-distribution). Note that both 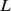 and 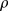 are functions of the network's architecture.

Given both 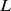 and 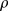, one can determine many properties of the network's equilibrium dynamics. For example, the probability that the 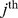 neuron fires at any given time (i.e., the steady-state firing-rate of the 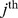 neuron) is given by
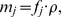
where 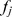 is a 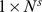 operator such that 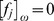, except for states in which 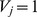, in which case 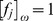 (i.e., 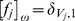). Similarly, the probability that the 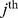 neuron fires at times 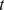 and 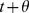, without firing at any intermediate times (denoted by 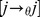) is given by
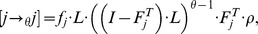
where 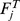 is a 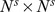 operator such that 
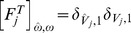
. The sensitivity of the network's firing-rates can be calculated via the 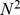 derivatives 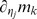. The reliability var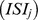 of the 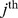 neuron in the network can be characterized by calculating the variance of 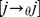 (considered as a distribution with respect to 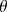).

Ideally, one might wish to determine how dynamic sensitivity (i.e., 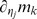) and reliability (i.e., var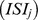) vary as functions of a network's architecture. Unfortunately, the explicit functional dependence of 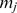 and 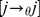 on architectural parameters (such as 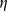, 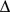, 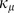, 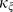) cannot be directly determined for most typical networks. However, it is possible to approximate these quantities by considering a weak-coupling expansion of 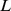 and 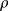 in terms of 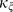.

If 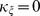, then each ORN-LNI pair is independent from the rest of the network, and the full state-evolution operator 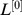 can be constructed by taking an operator-direct-product of the various 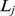, where 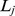 is the 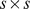-dimensional state-evolution operator associated with the 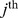 ORN-LNI pair shown in Eq. 16 (note that if 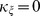, then each 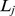 is independent of all other neurons). Similarly, if 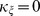 the full equilibrium-distribution 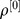 can be constructed by taking the product of the various 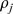, where 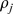 is the 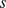-dimensional equilibrium-distribution of the 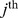 ORN-LNI pair (note that 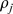 is the eigenvector of 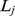 with eigenvalue 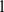). Both 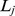 and 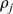 only depend on 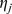 and 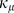. The sensitivity and reliability for this uncoupled 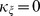 network can be determined simply by computing the sensitivity and reliability for individual (uncoupled) ORN-LNI pairs.

If 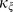 is small, then the network's full state-evolution operator 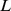 is no longer a direct product of the 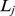 (and 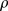 is no longer a product of the 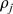). Nevertheless, by taking a Taylor-expansion of 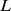 in terms of 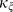 (around 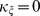) one can approximate 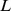 and 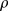 via a series
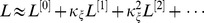


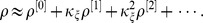
It can be shown that the 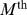-order terms in these series (corresponding to 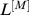 and 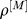) incorporate subnetworks of the original network spanning up to 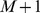 ORN-LNI pairs [24], [25]. Specifically, the 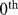-order terms capture the equilibrium dynamics of each single ORN-LNI pair in the absence of the rest of the network. The 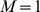 terms capture the first-order corrections associated with a single presynaptic-inhibitory connection of the form 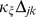. The 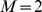 terms capture both the second-order corrections associated with a single presynaptic-inhibitory connection (of the form 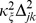), as well as the second-order corrections associated with 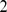 presynaptic-inhibitory connections (of the form 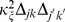). In the main text (Fig. 7), we have grouped the 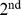-order terms corresponding to only 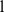 presynaptic-inhibitory connection with the 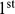-order terms associated with that connection. For example, when presenting the term associated with the subnetwork 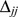, we implicitly include both the 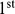-order term proportional to 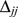, as well as the 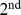-order term proportional to 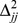. When presenting the term associated with the subnetwork 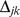, we implicitly include both the 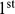-order term proportional to 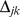, as well as the 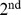-order term proportional to 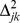.

Using the series-expansion for 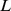 and 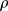, one can compute a series-expansion for many quantities of interest (e.g., firing rate 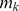, autocorrelation 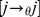, sensitivity 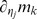 or reliability var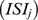) in terms of subnetworks of the original network. One attractive feature of this approach is that the formal series-expansion can be constructed without specifying the connectivity matrix 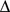. The terms of the series expansion can then be analyzed to determine which connectivity matrices will give rise to various dynamic phenomena. As an example, the terms 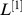 and 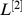 in the series expansion for 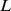 can be written as:
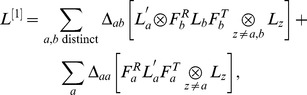


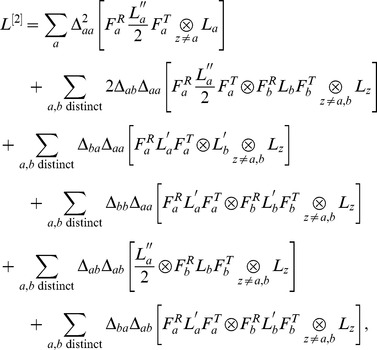
where we use the notation that 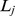 is equal to the 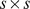 single-neuron operator shown in Eq. 16, with 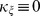, and 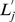 is the derivative of 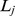 with respect to 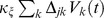 (the coupling parameter which appears in 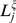). We also use the operator
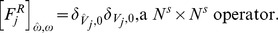
In the above representation of 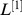 and 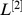, we use 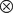 to denote an operator-direct-product, and 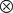 to denote an accumulation of operator-direct-products (analogous to the use of ‘
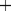
’ and ‘
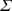
’ respectively).
